# Microarray Analysis of Copy Number Variants on the Human Y Chromosome Reveals Novel and Frequent Duplications Overrepresented in Specific Haplogroups

**DOI:** 10.1371/journal.pone.0137223

**Published:** 2015-08-31

**Authors:** Martin M. Johansson, Anneleen Van Geystelen, Maarten H. D. Larmuseau, Srdjan Djurovic, Ole A. Andreassen, Ingrid Agartz, Elena Jazin

**Affiliations:** 1 Department of Organismal Biology, EBC, Uppsala University, Uppsala, Sweden; 2 Laboratory of Socioecology and Social Evolution, Department of Biology, KU Leuven, Leuven, Belgium; 3 Forensic Biomedical Sciences, Department of Imaging and Pathology, KU Leuven, Leuven, Belgium; 4 Department of Medical Genetics, Oslo University Hospital, Oslo, Norway; 5 NORMENT, KG Jebsen Centre for Psychosis Research, Department of Clinical Science, University of Bergen, Bergen, Norway; 6 NORMENT, KG Jebsen Centre for Psychosis Research, Division of Mental Health and Addiction, Oslo University Hospital & Institute of Clinical Medicine, University of Oslo, Oslo, Norway; 7 Department of Psychiatric Research, Diakonhjemmet Hospital, Oslo, Norway; University of Pisa, ITALY

## Abstract

**Background:**

The human Y chromosome is almost always excluded from genome-wide investigations of copy number variants (CNVs) due to its highly repetitive structure. This chromosome should not be forgotten, not only for its well-known relevance in male fertility, but also for its involvement in clinical phenotypes such as cancers, heart failure and sex specific effects on brain and behaviour.

**Results:**

We analysed Y chromosome data from Affymetrix 6.0 SNP arrays and found that the signal intensities for most of 8179 SNP/CN probes in the male specific region (MSY) discriminated between a male, background signals in a female and an isodicentric male containing a large deletion of the q-arm and a duplication of the p-arm of the Y chromosome. Therefore, this SNP/CN platform is suitable for identification of gain and loss of Y chromosome sequences. In a set of 1718 males, we found 25 different CNV patterns, many of which are novel. We confirmed some of these variants by PCR or qPCR. The total frequency of individuals with CNVs was 14.7%, including 9.5% with duplications, 4.5% with deletions and 0.7% exhibiting both. Hence, a novel observation is that the frequency of duplications was more than twice the frequency of deletions. Another striking result was that 10 of the 25 detected variants were significantly overrepresented in one or more haplogroups, demonstrating the importance to control for haplogroups in genome-wide investigations to avoid stratification. NO-M214(xM175) individuals presented the highest percentage (95%) of CNVs. If they were not counted, 12.4% of the rest included CNVs, and the difference between duplications (8.9%) and deletions (2.8%) was even larger.

**Conclusions:**

Our results demonstrate that currently available genome-wide SNP platforms can be used to identify duplications and deletions in the human Y chromosome. Future association studies of the full spectrum of Y chromosome variants will demonstrate the potential involvement of gain or loss of Y chromosome sequence in different human phenotypes.

## Introduction

Multiple genome-wide analyses of copy number variants (CNVs) have been fruitful in the search of genes containing genomic variations causing human disease [1]. Although these tests are supposed to search throughout the genome, the Y chromosome has been excluded from most investigations [2]. Studies of CNVs on the Y chromosome are not only of interest for fertility research [2, 3], but are also relevant for cancer [4, 5], genealogy [6, 7], forensic medicine [8, 9], phylogeography and population genetics [10, 11], and the determination of sex differences in brain and behaviour [12, 13]. The exclusion of the Y chromosome has been due to the idea that the highly repetitive sequence structure and haploid state would hamper analysis with current genome-wide CNV methods [14]. The Y chromosome is the shortest and least gene rich of all human chromosomes. It has lost many genes during evolution, and includes 78 genes in modern humans. The sequence is divided into three specific regions named X transposed, degenerative, and ampliconic [15, 16]. The X transposed region has high homology to the X chromosome, and contains two single copy genes. The degenerative region exhibits higher divergence to its X homologous part and contains 16 genes. The ampliconic region shows the least similarity to the X chromosome and differs distinctively from the previously mentioned regions by the presence of eight palindromes, each varying in size and gene content [17].

The high homology of a segment of the p-arm on the Y chromosome (p11.2) to the corresponding part on the q-arm on the X chromosome (q21), together with the enrichment in repetitive and palindromic sequences elsewhere on the Y chromosome, make studies of CNVs on the Y chromosome a challenging task. Traditionally, methods such as PCR, qPCR and FISH have been used to dissect CNVs in specific regions of the Y chromosome [18–20]. These methods range from wide detection range (FISH), to discrimination of short segments (PCR and qPCR), and the main drawback is that they only analyse the presence or absence of CNVs in relatively short genomic regions, while the rest of the Y chromosome remains unexplored [21]. In this study, we used intensity signals after hybridisation to a Genome-wide human SNP array 6.0 from Affymetrix (Affymetrix 6.0 array). The fact that these arrays were not frequently used for the analysis of Y chromosome variants is surprising, since the male specific region (MSY) of the Y chromosome is well represented by 8179 probes, of which 288 are single nucleotide polymorphisms (SNP) probes and 7891 are copy number (CN) probes. The distribution of the probes covers most of the Y chromosome sequence, including palindromic areas. The pseudo autosomal regions (PAR) contain genes which have been found to have implication in pulmonary aveolar proteinosis [22], bipolar affective disorder, asthma [23] and short statue phenotype [24]. Recent evidence shows that duplications of *XG* and *GYG2* genes on PAR1 can be traced within at least two Y-chromosomal subhaplogroups, namely subhaplogroups I2a-P37.2 and R1b-P312* [25]. The PAR1 is covered by 426 SNP/CN probes with an average distance between the probes of 5.9kb. PAR2 has a lower coverage by only 42 SNP/CN probes with an average distance of 6.4kb. Copy number variant detection within PAR regions is indeed possible but several genes such as *CRLF2*, *CSF2RA* and *VAMP7* are not covered by any probes while the *IL3RA* and *IL9R* are represented only by one and two probes respectively. The probability of CNV detection within *IL3RA* and *IL9R* is minimal unless the CNV occurs over a larger region that will also include probes in these genes as well. Some small regions with highly repetitive sequence are not well represented by SNP/CN probes, including the *TSPY* multicopy gene region on the p-arm, and some amplicons in the AZFc region such as: u2, b1, b2, g1, r1, r2, r3 and g3.We here show that these arrays can be used to detect most of the previously known as well as many novel deletions and duplications in the Y chromosome.

## Materials and Methods

### Samples

We first analysed 271 Norwegian males previously included in a larger sample set used to investigate genome-wide associations with brain morphology, schizophrenia and bipolar disorder [26–28]. Affymetrix Genome Wide Human SNP Array 6.0 CEL files were available for re-analysis from their previous investigations that did not include the analysis of the Y chromosome. The 271 Norwegian males included 113 healthy controls and 158 cases, of which 57 were diagnosed with bipolar disorder, 65 with schizophrenia or schizoaffective disorder, and 36 had other diagnoses including depression and non-specified psychosis. Genomic DNA was available for all the 271 males. All the procedures performed in the current study were in accordance with the Declaration of Helsinki and all human DNA samples from the Norwegian population were collected after signed informed consent was obtained. Ethical permission for this work was from the Norwegian Ethics Committee (IRB number 2009/2485).

### Expansion of data

We also expanded our dataset by including Affymetrix Genome-Wide Human SNP Array 6.0 CEL files obtained from different populations available at the NCBI GEO Datasets [29]. These samples are referred to as “extended dataset”. The accession numbers for each dataset are as follows:

#### GSE21661

It includes data from 31 Tibetan samples from a study of high-altitude adaptation in Tibet [30]. 11 males were included in our study.

#### GSE29851

This is a genetic analysis of ancestry in Bolivian and Totonac populations of the New World [31]. It includes 24 Mesoamerican Totonac and 23 South American Bolivian males, all of which were included in our analysis.

#### GSE30481

A population study of copy number variations in China that did not include Y chromosome analysis [32]. From the total of 155 individuals, 79 males were included in our study.

#### GSE18333

An association study in which normal prostate tissue was compared to cancer prostate tissue. Samples were from Western countries and China [33]. 44 samples were included in our study.

#### GSE23201

Genome-wide CNV association study for schizophrenia in which the Y chromosome data was not included in the original analysis [34]. From the total of 735 individuals, 415 males (151 cases and 264 controls) were included in our study.

#### GSE23636

A genome-wide SNP investigation of Ashkenazi Jewish population structure [35]. From 471 individuals, 236 males were included in our study.

#### GSE15826

A study investigating sporadic motor neuron disease in patients and controls. Of 164 samples, 77 males were included in our study.

#### GSE13429

A study searching for CNVs in individuals with early onset colorectal cancer [36]. From 10 patients that were screened by Affymetrix 6.0 array, only 5 were males and therefore included in our study.

In addition to the NCBI GEO Datasets, we included data from two additional websites:

#### HapMap 3

(http://www.sanger.ac.uk/resources/downloads/human/hapmap3.html#t_download). This set included samples from 683 males and 714 females [37]. Females, individuals for which sex could not be determined and samples from data sets GIGAS, SCALE and SHELF were not included. Therefore, a total of 510 male samples were included in our study.

#### CG-SER-426

(http://www.cangem.org/CG-EXP-175/). Genome-wide association study of patients with developmental disorders. The Y chromosome was excluded from the analysis. From a total of 35 cases, 23 males were included in our study [38].

In total, 1718 CEL files from males were included in our study (S1 Table).

### Copy number variation analysis

Re-analysis of CEL files for CNV detection on the complete data set (1718 individuals) was done using Affymetrix Genotyping Console Software 4.1.3.840 (GTC). Copy Number/LOH analysis was performed using the Regional GC Correction setting and samples were normalized against GenomeWideSNP_6.hapmap270.na33.r1.a5.ref reference samples. The GenomeWideSNP_6.na33.annot.dn was used as annotation reference model. The segment report analysis was done with the following settings: minimum number of markers per segment set to 3, minimum genomic size of a segment (kbp) set to 5 and including 100% of the segments that overlap with known CNV regions. Since low stringency settings were used for maximum detection of possible CNVs, many false positive segments were marked in regions of low probe content. These false positives were curated manually after visual inspection of the plots produced by GTC. The results were exported as tab delimited text files containing the Log 2 ratio values for the Y chromosome, chromosomal position and the dbSNP RS ID values for graphical representation and additional analysis.

### SNP/CN probe assignment to ampliconic and palindromic regions and CNV type determination

To assign the SNP/CN probes included in the array to different palindromic and ampliconic sequences in the Y chromosome, we first selected from the literature sequence-tagged site (STS) markers previously used to define the limits of palindromic and ampliconic sequences. Markers sY1312 and sY1304 delimited P7, markers sY1275 and sY1276 for P6, sY1264 and sY1283 for P5, sY1283 and sY1277 for P4, sY1315 and sY1259 for IR2, sY142 for distal end of U1, sY1259 and sY1191 for P3, sY1191 and sY1192 for U3, sY1291 and sY1125 for gr1, sY1125 and sY1054 for b3, sY1054 and sY1206 for Y1, sY1206 and position of BPY2 for g2, sY254 and the position of DAZ3,4 genes for r3 and r4, sY1206 and sY1054 for Y2, sY1054 and sY1125 for b4, sY1054 and 1201 for gr2 [16, 21, 39–42]. Then, we extracted position information for all the above listed markers from the UCSC genome browser (hg19). Finally, All SNP/CN probes within the start and stop positions for the limiting STSs were assigned to each particular palindrome/amplicon. For all 235 individuals with detected CNVs, we plotted the intensity signals for all MSY probes using colour codes to represent different palindromic and ampliconic sequences according to Repping et al. [41]. Copy number state of palindromic or ampliconic regions were used to assign each CNV pattern to different subtypes following the nomenclature by Repping et al [41]. One example for each type of CNV is shown in (S1 Fig).

### PCR verification of CNVs

Samples from individuals in which SNP array analysis detected CNVs were subjected to molecular verification by PCR. Primers that amplify sequences included in Region specific Sequence Tagged Sites were selected from the NCBI Probe database (http://www.ncbi.nlm.nih.gov/probe) as follows: primers for sY1191 and sY1192 amplify a segment included in the U3 region and sY1291 primers map to the boundary between r2 and the gr1 segment. The reaction mix contained: 2.5μl PCR Buffer (Thermo Scientific), 0.5μM of each of the primers, 0.2μM for each of the dNTPs, 0.5 units of *Taq* DNA Polymerase (Thermo Scientific) and 50ng of genomic DNA in a final volume of 25μl. PCR amplification was carried out on a PTC-100 thermocycler (MJ Research) with an initial heating at 94°C for 90 seconds, followed by 36 cycles of 94°C for 30 seconds, annealing at 60°C (sY1191), 63°C (sY1192) or 62°C (sY1291) for 35 seconds, 72°C for 60 seconds and final extension at 72°C for 5 min. Products were separated by electrophoresis on 1.75% agarose gels using 7μl or product mixed with 3 μl Gel Orange. Gels were stained using GelRed (1μg/ml of 0.5M TAE Buffer) and photographed under UV light using Gel DocTM XR (Bio-Rad, Hercules, CA, USA). Female samples we amplified as controls for each set of primers.

### Real-time PCR analysis

Genomic DNA for individuals with P6 duplication, male controls without CNVs and samples from females were diluted to a final concentration of 5ng/μl. All samples were run in triplicates. Two STS markers, sY1081 and sY933 were selected because of their location within the P6 duplicated region, while RH38681 was chosen because of its location upstream from this CNV variant. Primer sequences for these markers were selected from the NCBI Probe database (http://www.ncbi.nlm.nih.gov/probe), and were controlled for appropriate product size and annealing temperature for qPCR experiments. The reaction mix included 10 μl Power SYBR Universal PCR Master mix (ABI), 1.25 μl for each of the primers (5 μM), 5 μl DNA (25 ng) and RNase-free water to a final volume of 25 μl. The real-time PCR amplification was carried out on a 7500 Real-time PCR system (Applied Biosystems) with initial heating at 94°C for 90 seconds, followed by 40 cycles of 94°C for 30 seconds, annealing at 61°C for 35 seconds, 72°C for 60 seconds and a final extension at 72°C for 5 min. A melting curve from 94°C to 55°C for 20min was run to control for the absence of primer dimers.

### Determination of Y chromosome haplogroups

The phylogenetic position of each Y chromosome, based on the 91 SNPs which are in both the Affymetrix 6.0 array and the most up-to-date Y-chromosomal tree [43] was determined with the AMY-tree algorithm [44, 45], which takes into account the missing Y-SNPs in order to retrieve the most accurate sub-haplogroup possible.

### Statistical analysis

Principal Component Analysis was performed using JMP, Version *11*. SAS Institute Inc., Cary, NC, 1989–2007. Data from 246 MSY specific SNPs together with the haplogroup assignment for each individual were used for the analysis using the standard (correlation) settings. SNP information was translated to numbers 1 to 4 for G, C, T and A. Missing values were given value of zero. Data from first and second eigenvectors were used to prepare the figures in the manuscript.

Pearson Chi-Square, Likelihood ratio and Fisher´s exact test were performed using the Statistical Package for Social Sciences Statistical Software release 21 (SPSS Windows Version 21, SPSS, Inc.).

## Results

### Affymetrix 6.0 arrays can be used for CNV detection on the Y chromosome

**Fig 1:**
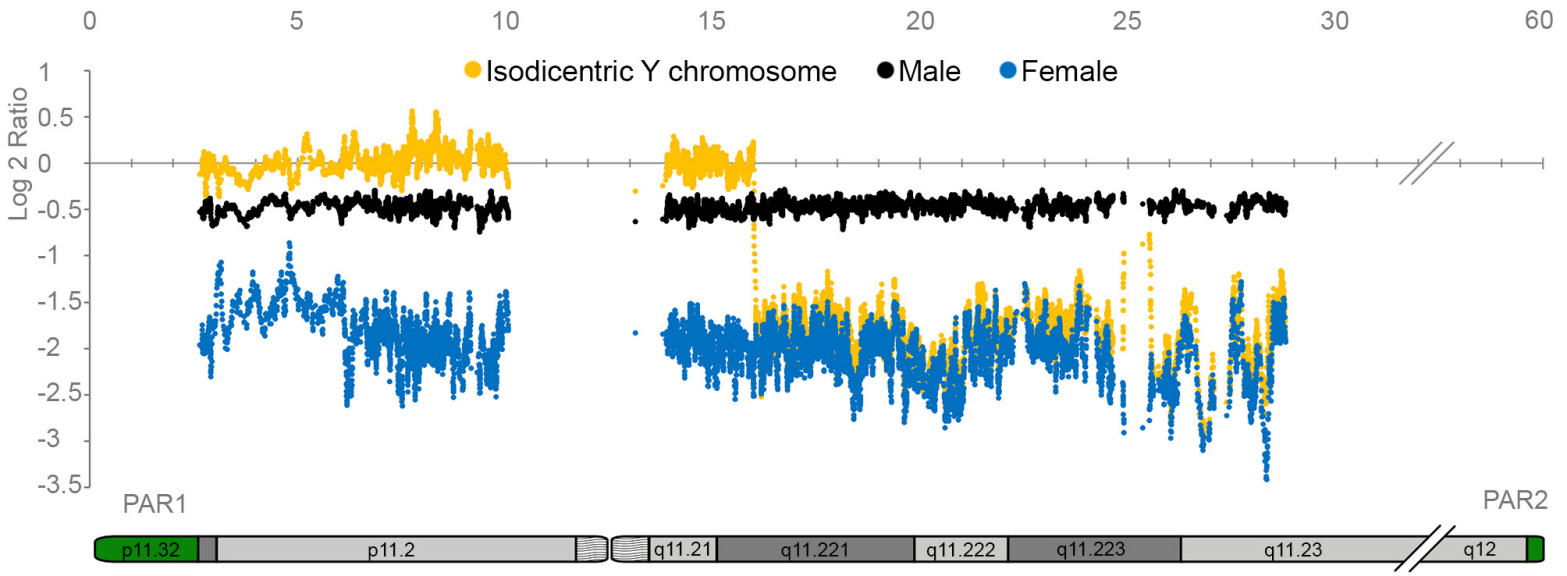
Comparison of Y chromosome SNP/CN probe intensities between a male, a female and a male with an isodicentric Y chromosome. The Y axis denotes the log ratio of the average signal intensities for each 20 consecutive SNP/CN probes, while the X axis denotes the genomic position on the Y chromosome, in million base pairs. The signals for a control male are represented in black, female signals are in blue and the signals for the male with an isodicentric Y chromosome are in yellow. Only probes corresponding to the male specific Y region are included.

To validate the use of Affymetrix 6.0 arrays for the detection of Y chromosome specific variants, we compared signal intensity ratios for all Y chromosome SNP/CN probes between a female, a male, and an additional male known to have an isodicentric Y chromosome [20]. Fig 1 shows the average intensity for each set of 20 consecutive SNP/CN probes.

In all cases, the average intensities of males were higher than for females. The total average for all 8179 Y chromosome SNP/CN probes in ten randomly selected males was -0.47 (Log 2 ratio), while the corresponding number for ten females was -2.02. Similarly, when we calculated the average intensity for each set of three consecutive SNP/CN probes in 61 females versus 25 males, most sets of probes presented large differences between females and males. However, three regions presented high background levels in females, with average intensities higher than -0.47 (S2 Table). The individual with an isodicentric Y chromosome has a deletion spanning most of the q-arm and a duplication of the remaining part of the chromosome (20). As expected, this individual had signal intensities similar to female in the deleted part of the Y chromosome, and about twice the intensities observed in males in the duplicated part, resulting in an average Log 2 ratio of 0.04 in this region. These results show that almost all (99.9%) Y chromosome SNP/CN probes can be used to detect deletions and duplications throughout the Y chromosome.

### CNV discovery in a Norwegian sample population

**Fig 2:**
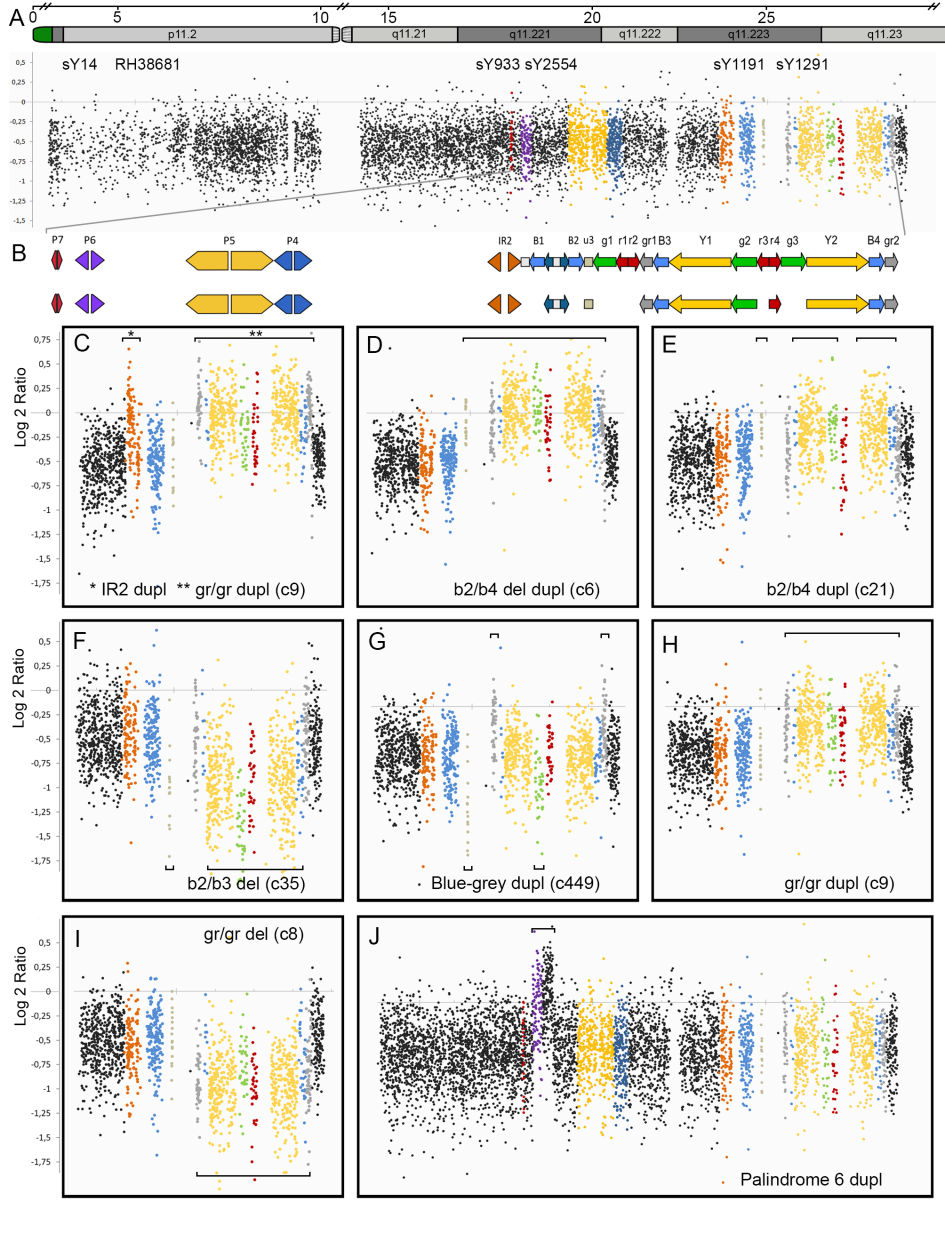
Y chromosome CNV discovery in a Norwegian population. A. Signal intensity plot (Log 2 ratio) for a control male without CNV variants. Signals from each of the 8179 probes are represented by one dot. B. Regions of the Y chromosome containing ampliconic and palindromic sequences are represented by colored arrows using the same nomenclature as in Repping et al. [41]. The same colors were used to represent the signal intensity of the corresponding regions in the rest of the figure. Not all ampliconic sequences are covered by SNP/CN probes in the array. The lower part of Fig 2B includes only detectable regions. C to I. Signal intensity plots (Log 2 ratio) for different type of CNVs discovered in a Norwegian population. The lower part of each subfigure shows the name of each CNV following the nomenclature used in Repping et al. [41]. Staples delineate the positions of duplications (on the top of the figure) or deletions (on the bottom). J. Signal intensity plot for a previously undescribed duplication hereby named P6 dupl.

We first analysed SNP array data obtained from 271 Norwegian males previously included in Norwegian genome-wide association studies of brain morphology, schizophrenia and bipolar disorder [26–28]. In this population we found 31 males exhibiting eight different CNV variants on the Y chromosome (Fig 2).

Four of these, including b2/b3 del (Fig 2F), blue-grey dupl (c449, Fig 2G), gr/gr dupl (c9, Fig 2H), and gr/gr del (Fig 2I), correspond to variations in the AZFc region that have been well characterized previously [46–48]. Two additional variants correspond to the b2/b4 region, including b2/b4 del dupl (c9, Fig 2D) and b2/b4 dupl (c21, Fig 2E). Similar duplications in the b2/b4 region have been found previously [49]. IR2 duplications (Fig 2C) have not been previously detected.

**Fig 3:**
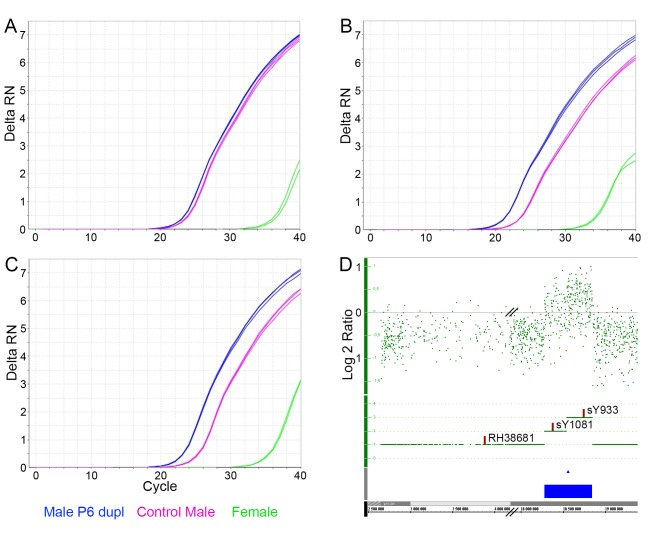
qPCR validation of the newly discovered P6 duplication. Amplification plots for a female (green), a control male (purple) and a male with P6 dupl. (blue) are shown for markers RH38681 (A), sY1081 (B) and sY933 (C). D. Intensity signal plot (Log 2 ratio) for an individual with P6 dupl. showing that markers sY1081 and sY933 are positioned within the duplicated region, while RH38681 is located outside.

In addition, the last variant was named Palindrome 6 duplication (P6 dupl), started at position 18.279.605 (UCSC genome browser hg19) and ended at position 18.843.147. This duplication has not been previously described (Fig 2J). In the Norwegian population, five individuals displayed this variant, two of which also had a CNV in the AZFc region, including one b2/b3 del and one gr/gr dupl. We confirmed detected variants using STS markers for PCR analysis. sY1191 and sY1192 did not amplify in individuals with b2/b3 del and blue-grey dupl while they produced amplification bands in individuals with gr/gr del. Marker sY1291 amplified positively in individuals with b2/b3 del and blue-grey dupl while it was negative for gr/gr del (data not shown). For confirmation experiments of the newly discovered P6 dupl. we used a qPCR approach including markers RH38681, sY1081 and sY933 (Fig 3).

The figure shows that markers sY1081 and sY933 had larger amplification signals in an individual with P6 dupl. compared to a control.

To increase the chance to detect as many CNVs as possible, we included in the screenings both healthy individuals as well as individuals with psychiatric diagnosis as described in the Methods.

### CNV discovery in an extended dataset

When we analysed the signal intensities of all Y chromosome SNP/CN probes in the extended data set obtained from 1447 individuals from different populations (described in Methods), we found 17 additional types of variants, resulting in a total of 25 different CNVs in the complete data set (S1 Fig). Of these, several have not been described previously. For example, we named one of the novel variants “blue-grey like duplication” based on its resemblance to the previously described blue-grey dupl c449. In both cases the gr amplicons are duplicated and the U3 region is deleted (compare Fig 2G with S1P Fig). The difference between them is that the Y1, Y2 amplicons are deleted and the r amplicons are duplicated in the newly discovered variant, while these changes are absent in the regular blue-grey dupl. Furthermore, the g amplicons are deleted to a greater extent in the new variant (S1P Fig). The second newly found CNV was named “P5 duplication” and includes a large group (n = 19) of similar but not identical duplications in the P5 region (S1E Fig). Interestingly, another novel pattern includes two duplications appearing in tandem. The first duplication is spanning the region upstream of P5, and the second duplication encompassing the region immediately after P4 (S1F Fig).

In general, we have found several additional novel duplications that cannot easily be discovered with traditional PCR analysis of STS markers (S1A, S1B, S1D, S1E, S1F, S1H, S1K, S1U, S1W and S1X Fig). In addition, we found three rare deletions in one individual each (S1B, S1C and S1J Fig).Although, it should be noted that we did not have access to the genomic DNA for the samples in the extended dataset and the therein observed CNVs could therefore not be verified by molecular methods.

### Determination of Y chromosome Haplogroups

Only 91 out of the 246 Y-SNPs present on the Affymetrix chip were informative in this study as the phylogenetic positions. The other 155 Y-SNPs were indeed unknown based on the used updated tree and other already published phylogenies. Extensive effort was put down to increase phylogenetic information in our study by exploring all open data sources and publications with full Y-chromosomal sequences in order to include any of the 155 remaining Y-SNPs into the phylogeny. As such, we explored all partial Y-chromosomal sequences from Hallast et al. [10], all full WGS samples mentioned in Van Geystelen et al. [43] and even all community-driven/experimental phylogenies on the ISOGG-website (www.isogg.org). Nevertheless, the phylogenetic position of the 155 Y-SNPs remained unknown or could not be resolved reliably and thus be informative in our study.

To determine haplogroups (HGs) in the complete collection of 1718 Y chromosomes, we applied the AMY-tree algorithm [44, 45] and the most up-to date Y chromosomal tree [43]. Individuals assigned to specific sub-haplogroups were backmerged to their corresponding haplogroup according to the information available at the minimal Y tree [38] (http://www.phylotree.org/Y/). S2 Fig shows that we found 9 main haplogroups in our dataset. In addition, three groups of individuals were assigned to internal nodes because the arrays did not contain SNP information for a particular haplogroup. For example, the arrays did not contain diagnostic SNPs for haplogroup N-M231. Forty individuals were not mutant for the SNPs of haplogroup O-M175 and they were mutant for the SNPs that define NO-M214. Therefore, they were assigned to node NO-M214 named NO-M214(xM175) in all analysis herein (S2 Fig). In other words, forty samples most likely belong to haplogroup N-M231, but we could not assign them to this haplogroup as there were no Y-SNPs describing N-M231 or one of its subhaplogroups included in the Affymetrix 6.0 arrays. Similarly, ten individuals were included in haplogroup F-M89(xF1329) as they were mutant for the SNPs of F-M89 but not for those of GHIJKLT-F1329, and the arrays did not include diagnostic SNPs for F2-M247 or its subhaplogroups. A third group of 29 individuals was included in haplogroup KLT-M9(xM526) as they were mutant for the SNPs of KLT-M9 but not for those of K-M526 and its subhaplogroups (S2 Fig).

For 38 individuals the determination of the haplogroup was ambiguous, in the sense that the algorithm suggested more than one possible HG. Additional 17 individuals did not have any sequence difference from the root sequence, thus could not be assigned to any HG. All these 55 individuals were excluded from further statistical analysis. As it can be observed in S2 Fig, three additional groups of individuals were assigned to internal nodes. These groups consisted of 16 individuals assigned to internal node DE-M145, 30 individuals in node P-P295 and 111 individuals in node IJ-M429. There was not enough supporting data to assign a more specific haplogroup, although this should be possible based on the SNPs included in Affymetrix 6.0 arrays. This suggests that information for SNPs important for haplotype determination was missing for these samples, and we removed these 157 individuals from PCA and CNV frequency analysis.

**Fig 4:**
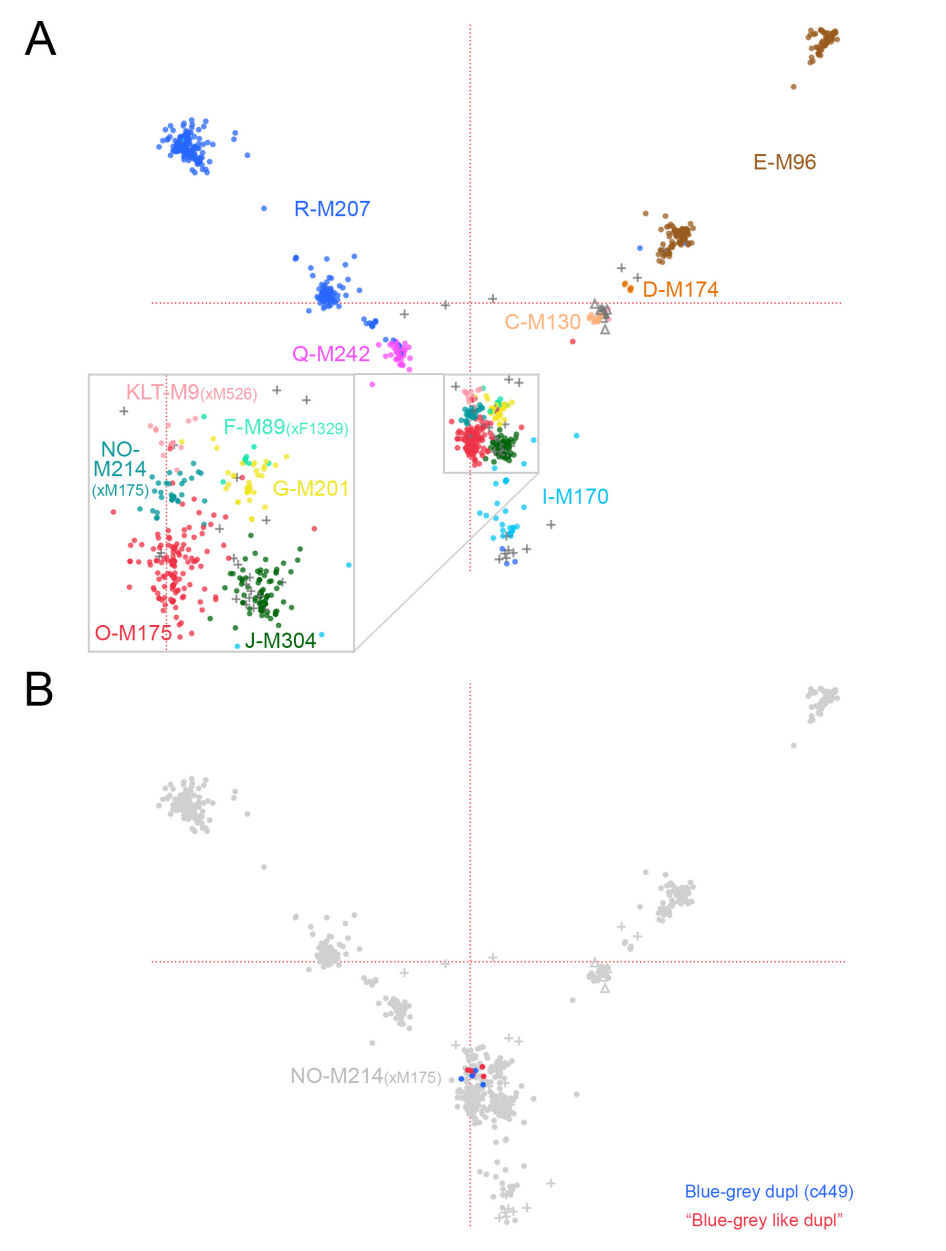
Principal component analysis of Y chromosome specific SNP variation between haplogroups. A. The figure shows the graphical representation of the first two eigenvectors after PCA analysis. Y-axis corresponds to the first vector explaining 24.1% of the variation and X-axis explains 13.4% of the remaining variation. Each dot represents the results from one individual and the colour represent each HG as denoted by letters in the figure. The plus symbols in black denote individuals for which HG determination was ambiguous. The black triangles denote individuals for which no HG could be assigned. B. The results from the individuals carrying the blue-grey dupl are represented in blue, while the results from individuals carrying “blue-grey like dupl.” are in red. All cases are included within the NO-M214(xM175) haplogroup. In total 11 individuals carry these variants (Table 1) but they are superimposed in the figure, due to high similarity between their HG.

Principal component analysis was performed as described in Methods with data from 1506 individuals belonging to 12 haplogroups. We found that the first and second eigenvectors explained 24.1% and 13.4% of the variability between groups. In general, the HGs are clearly segregated by this analysis, particularly R-M207, E-M96 and I-M170. Haplogroups NO-M214(xM175), KLT-M9(xM526), F-M89(xM1329), G-M201, J-M304 and O-M175 exhibit larger similarity between each other, but they can still be distinguished (Fig 4A).

### CNV distribution among haplogroups

When we linked all the discovered Y chromosome variants with the corresponding HG we observed uneven distributions. Table 1 shows ten variants for which the distribution was significantly associated with one or more haplogroups as determined by Pearson Chi-Square, Likelihood ratio and Fisher´s Exact Tests.

**Table 1:** Distribution and frequency of CNV patterns significantly overrepresented within haplogroups.

Haplogroup	Nr of ind.	Q-arm dupl+U3 del	P5 dupl	P4 dupl	P3 dupl	b1/b3 del c3	b2/b3 del c35	Blue-grey dupl c449	Blue-grey like dupl	gr/gr dupl + distal dupl	gr/gr del c8
**C-M130**	24	1* (7.8)				1* (3.7)	1				1
**D-M174**	6										5* (14.9)
**E-M96**	339		3		18 (7.6)						3
**F-M89(xM1329)**	10			2 (6.5)							
**G-M201**	80		8 (7.0)			1					
**I-M170**	40										1
**J-M304**	242		1							13* (8.3)	4
**KLT-M9(xM526)**	29			4 (7.5)							2
**NO-M214(xM175)**	40						27* (31.1)	4* (12.1)	7* (16.1)		
**O-M175**	162		3	3		2* (2.5)	2				6
**Q-M242**	56		4 (3.9)	4 (5.1)							1
**R-M207**	478				1						4
**Ambiguous**				1			1				2
**Pearson Chi-Square p-value**		2.7E-02	1.6E-04	1.1E-05	2.6E-04	6.8E-02	8.0E-45	3.2E-04	1.3E-06	1.2E-03	7.0E-10
**Likelyhood ratio p-value**		2.7E-02	nd	2.0E-10	nd	1.8E-02	3.5E-51	3.8E-06	1.0E-10	2.5E-08	nd
**Fisher´s Exact Test p-value**		2,7E-02	1,0E-06	6,4E-10	nd	2,0E-02	1,0E-49	1,7E-05	1,2E-09	5,0E-06	nd
**Sum of CNV type**		1	19	13	19	4	30	4	7	13	27
**% of CNV type**		0.07%	1.26%	0.86%	1.26%	0.27%	1.99%	0.27%	0.46%	0.86%	1.79%

The table shows the distribution of CNV patterns among haplogroups for ten variants that showed overrepresentation in one or more haplogroups. Ambiguous individuals, for which haplotype determination was not possible, are shown in the table for completeness, but they were not included in the statistical analysis. The p-values after Pearson Chi-Square analysis, likelihood ratio and Fisher’s exact test are shown at the bottom of the table, together with the total amount of each CNV type. The % frequency is derived from CNV type observations divided by 1506 individuals for which haplogroup could be determined. The stars mark values that are significant with the standard residual indicated in parenthesis. (Ind.) individuals, (dupl) duplication, (del) deletion and (nd) not done.

**Fig 5:**
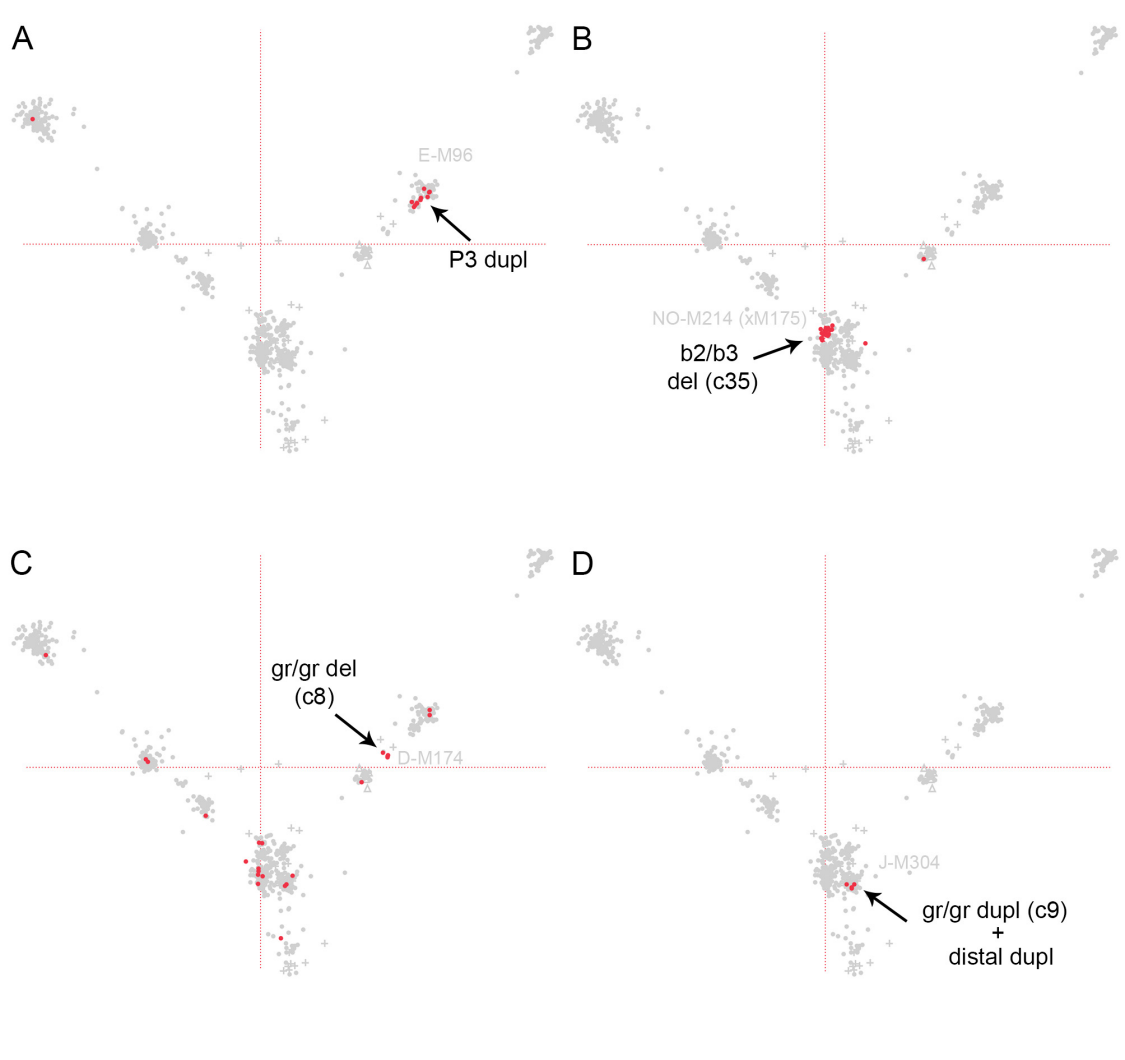
Distribution of CNV patterns that were significantly overrepresented in one haplogroup only. Each part of the figure shows the graphical representation of the first two eigenvectors after PCA analysis A. The figure shows PCA values for individuals with P3 dupl. significantly overrepresented in haplogroup E-M96. B. Individuals with b2/b3 del. significantly overrepresented in haplogroup NO-M214(xM175). C. Individuals with gr/gr del. (c8) significantly overrepresented in haplogroup D-M174. This CNV is also present in other haplogroups. D. Individuals with gr/gr dupl. (c9) + distal dupl. significantly overrepresented in haplogroup J-P256.

For example, blue-grey dupl and the newly discovered “blue-grey like dupl” were exclusively present in individuals belonging to NO-M214(xM175) (Table 1, Fig 4B). The b2/b3 del was also highly overrepresented in NO-M214(xM175) individuals, with 27 of 40 Y chromosomes containing this variant (p = 8E-45, Table 1, Fig 5B). P3 duplications and “gr/gr dupl + distant dupl” were in most cases present in only one haplogroup each, namely E-M96 and J-M170 (p = 2.6E-04 and p = 1E-03 respectively, Table 1, Fig 5A and 5D).

The gr/gr deletion was distributed among many haplogroups but it was significantly overrepresented within haplogroup D-M174, with five of six individuals containing this variant (p = 7E-10, Fig 5C). P5 dupl, P4 dupl. and b1/b3 del. were all overrepresented in more than one haplogroup (Table 1 and S3 Fig). The first CNV variant listed in Table 1 corresponds to a single occurrence of a q-arm duplication together with an U3 deletion observed in one male belonging to haplogroup C-M130 (S1B Fig). Despite the low frequency of this CNV, the small amount of Y chromosomes belonging to haplogroup C-M130 (24 individuals) makes this observation significant (p = 0.027 by two sided Fisher´s exact test). The q-arm duplication spanned exons 34–44 including the 3´UTR in the *USP9Y* gene (positions 14939249–14974460 according to hg 19, size 35kb, including 21 SNP/CN probes). The *USP9X* region did not exhibit any variations in this individual.

The remaining 14 variants were not significantly overrepresented in any haplogroup (S3 Table). However, it can be noted that the newly discovered P6 duplications were present in individuals classified to R-M207 and one individual in NO-M214 haplogroup. Interestingly, the “Prior P5 post P4” duplications and P3 deletions were only present in haplogroup E-M96. The gr/gr duplications were widely distributed among eight haplogroups. This CNV had a total frequency of 2.52% among all haplogroup assigned individuals (1506), making it the most common pattern in this study.

S3 Table also lists a rare large deletion of the q-arm (782 kb, including 338 SNP/CN probes, position ChrY: 14.448.577–15.230.545) in a single individual, completely removing four genes, *GYG2P1*, *TTTY15*, *USP9Y* and *DDX3Y* (S1C Fig). The table also includes five different p-arm duplications. Of these, two deserve to be mentioned. The first case duplicates a 160.216 bp region affecting the entire *AMELY* gene and the first two exons of *TBL1Y*, including both promoters for these genes (S1A Fig). The second case duplicates a region of 148 kb (positions 4.654.108–4.802.101 according to hg19), upstream of the *PCDH11Y* gene, not affecting the promoter region. We also studied the percentages of individuals with Y chromosome CNV in each haplogroup (Table 2). In the data set including 1506 Y chromosomes for which HG information was available, 14.7% exhibited some kind of CNV but the distribution was very uneven between haplogroups. Most notably, 97.5% of individuals from haplogroup NO-M214(xM175) carried a CNV. Haplogroup D-M174 indicated high prevalence of gr/gr del (83.3%) but it should be noted that this haplogroup had the lowest amount of samples in this study, only 6 individuals.

**Table 2:** Sample distribution and frequency of any CNV in each haplogroup.

Haplogroup	Nr of individuals	Percentage of total individuals included	Individuals with CNV	Percentage of CNVs in HG
**C-M130**	24	1.6%	5	20.8%
**D-M174**	6	0.4%	5	83.3%
**E-M96**	339	22.5%	44	13.0%
**F-M89(xM1329)**	10	0.7%	2	20.0%
**G-M201**	80	5.3%	12	15.0%
**I-M170**	40	2.7%	7	17.5%
**J-M304**	242	16.1%	30	12.4%
**KLT-M9(xM526)**	29	1.9%	7	24.1%
**NO-M214(xM175)**	40	2.7%	39	97.5%
**O-M175**	162	10.8%	25	15.4%
**Q-M242**	56	3.7%	11	19.6%
**R-M207**	478	31.7%	34	7.1%
Total	1506	100.0%	221	14.7%

From a total of 1718 individuals, 1506 could be assigned to specific haplogroups based on a limited set of phylogenetically informative Y-SNPs within the Affymetrix 6.0 arrays. The table shows the distribution among haplogroups for these individuals, as well as the frequency of CNVs within each haplogroup. The highest frequency (95%) of CNVs was found among individuals of NO-M214(xM175) haplogroup.

### Assessment of CNV presence in patient vs control samples

To investigate if the males with psychiatric diagnosis in the Norwegian population differed from the controls in CNV frequencies, we calculated the total frequency of variants in each group and found that 10.6% of healthy individuals included CNVs, while 13.8% of patients with schizophrenia and 14% of bipolar subjects included variants. These differences were not significant according to Fisher’s test. In males from the GSE23201 study, 13 out of the 139 included schizophrenia cases exhibited a CNV (8.6%) while 21 out of 132 control males carried a CNV (15.9%) resulting in no significant difference between these groups (p = 0.14). A Fisher´s exact test could not detect any significant (p = 0.26) overrepresentation of CNVs in any of the six HGs (E-M96, G-M201, I-M170, J-M304, KLT-M9(xM526) and R-M207) that were present in the 139 schizophrenia affected males. Neither did any of the HGs exhibit any overrepresentation in cases or controls (p = 0.515). Thereby, we could not detect any association between Y chromosome variation or haplogroup type and prevalence of schizophrenia in males. The same conclusions apply for the Norwegian population with corresponding p values of: 0.516, 0.097 and 0.656 all derived from Fisher´s exact test (bipolar, schizophrenia and psychosis merged). Since most of the CNVs occur in the AZFc region which contains fertility genes, then the probability that those CNVs might be involved in contribution to psychiatric disease becomes highly unlikely. In general, the CNVs on the p-arm and the upper q-arm would be more probable candidates for association to psychiatric diseases since those regions hosts *PCDH11Y* and *NLGN4Y*. Both of these genes expressed in the central nervous system and are involved in cell adhesion and synapse formation respectively.

## Discussion

We show here that Affymetrix 6.0 arrays can be used for high resolution detection of Y chromosome specific deletions and duplications, and therefore genome-wide analysis of copy number variants using this type of arrays should include the analysis of Y chromosome. In addition, MSY specific SNPs included in these arrays can be used to determine with certainty most major Y chromosome haplogroups. An analysis of a large collection of 1718 individuals allowed detection of many previously known, as well as many novel Y chromosome copy number variants. Most important, we found that several of these CNVs are significantly overrepresented in specific haplogroups.

### Y chromosome CNV detection

The Affymetrix 6.0 arrays are suitable for studies of Y chromosome variants not only because of the high coverage and wide distribution of 8179 SNP/CN probes throughout the Y chromosome, but also because we show that most of these probes (excluding those listed in S1 Table) could distinguish the signal intensities between a male, a female, and an individual with isodicentric Y chromosome. Regions not covered by any probes include the centromere and some of the amplicons in the AZFc region, including r1, r2, g1 and g4. However, the analysis of the remaining amplicons can be used to evaluate copy number state of the missing amplicons, thereby allowing determination with high resolution of the subtype of CNV according to the nomenclature by Repping et al [41]. Unfortunately, the region containing an array of *TSPY* genes is represented by only three probes and therefore cannot be used for CNV detection. Copy number variations in this region have been previously associated with male infertility or prostate cancer [50, 51].

### Discovery of Y chromosome CNVs

We detected 25 distinguishable CNV patterns in 242 out of 1718 surveyed Y chromosomes. Therefore, 14.1% of males carried one or more CNVs. Of these 9.0% corresponded to individuals that had duplications, 4.5% individuals had deletions and 0.6% had both types of variants. A large population analysis combining deletions and duplications in the Y chromosome has not been done previously, but a large survey of deletions has been done in 20.000 males [52]. This study found that 3.7% of the individuals carried any of the six deletions analysed. When we added the percentages of the corresponding deletions in our study the result was 4.1%. This shows that our SNP array analysis has similar power to detect deletions. A novel observation in our study is that duplication events occur at more than twice the frequency of deletions. Most of the duplications we discovered are novel, although duplication of the AZFc region and the Palindrome 5, which are the most commonly found in our study, have been detected previously (but not assigned to specific variants) by the first generation of comparative genomic hybridization arrays [53, 54].

In our study, both deletions and duplication of the AZFc region were common. In contrast, in the palindromic regions P6, P5, P4 and IR2 we found almost exclusively duplications. One possibility for why we observe only duplications in P6 and P4 palindromes, could be the fitness effect. A loss of the genes contained by these palindromes might have severe impact on the carrier and thus not being propagated or seen very often. Although, the low gene content in P6 contradicts the fitness argument, while the P4 gene content (*HSFY2* and *TTTY9A*, *NCRNA00185*, *CD24* and *TTTY14*) on the other hand supports it. Especially, as the *TTTY14* is known to be expressed in the male brain during early foetal development [55]. In the work of Hallast et al. [40], the authors notes that there is a ncRNA (107bp) residing in the P6 structure. Upon closer inspection that snRNA (RNU6-109P1) is classified as a pseudogene. Also, AC053516.1 can be found within P6 and this 111bp sequence is classified as miRNA (UCSC genome browser hg 19). After all, the main function of a palindromic structure is to protect the sequence from degradation by gene conversion [56]. High conservation of the sequence in P6 between human, chimp and gorilla speaks in favor of functionality of the region [40]. Yet another possible explanation to the overrepresentation of duplications in the palindromic regions could be that the surrounding sequences of the palindrome borders might be enriched for VNTRs or different breakpoint sequence motifs which are mainly associated with duplications as described by Conrad et al. [57]. Two other mechanisms that might play a role in the skewed distribution could be Non-homologous end joining and Microhomology-mediated break-induced repair. Nevertheless, in the light of the data generated by Conrad et al. [57], the probability for the latter mechanisms being the key players in generation of duplication overrepresentation is low.

Another aspect that might influence the observed overrepresentation of duplications in the entire dataset might be the composition of HGs included. For example the HG E-M96 exhibits an exaggerated ratio of dupl to del of 3.8:1. Finally, our study unlike most others, investigates almost the entire MSY with probes spanning both intronic and exonic regions. The possibility of detecting CNVs in the intronic regions might lead to skewed ratio compared to other studies which focus on CNV detection within exonic sequences.

### CNVs overrepresented in specific haplogroups

Despite the relatively low amount of phylogenetically informative Y chromosome SNPs (n = 91) in the arrays, we were able to distinguish between most known major haplogroups, as recently described by a next generation sequencing approach [10] and also in [58]. When we studied the haplogroup distribution of the 24 detected CNV patterns, we found that ten of them were significantly overrepresented in one or more haplogroup. Of these, the most prominent result was the high frequency (67.5%) of b2/b3 del (c35) in haplogroup NO-M214(xM175). As explained in the results section, this haplogroup is most probably equivalent to N-M231. Therefore, we confirm the high frequency of b2/b3 del in haplogroup N-M231 observed previously [41, 46, 52]. Interestingly, two additional CNV patterns were detected only within this haplogroup. These include the blue-grey dupl. (c449) and a novel pattern that we denoted “blue-grey like dupl” not previously described in the CNV predictions by Repping et al [41]. These variants were found in several of our population data sets, including the Norwegian, Chinese, Tibetan and HapMap3 populations. The fact that the blue-grey dupl was found only in NO-M214(xM175) individuals supports the model according to which this variant originates as a rearrangement of b2/b3 deletion [46]. The high similarity between c449 and the newly discovered pattern suggest that this novel variant originated from an individual with c449 pattern in which deletion of Y1/Y2 and G amplicons, and a duplication of gr1/gr2 and r1/r2 occurred. Taken together the three CNVs described above, 95% of NO-M214(xM175) assigned individuals contained one of these variants, rendering NO-M214(xM175) to be the most CNV rich haplogroup. The relatively large proportion of individuals containing Y chromosome CNV (13.9%) found when all 1718 individuals were considered, is not largely affected by the results in NO-M214(xM175) individuals, since 12.3% of individuals with CNVs remain upon removal of NO-M214(xM175) individuals from 1506 individuals with assigned HG. Indeed, in this case the difference between duplications (9.4%) and deletions (2.9%) becomes even larger, with more than three times duplications than deletions in this dataset.

Of the remaining seven CNVs significantly overrepresented in specific haplogroups, two of them were almost exclusively present in individuals of one haplogroup each. Indeed, the P3 duplication was found in 18 individuals of haplogroup E-M96 and only once in haplogroup R-M207. Similarly, the “gr/gr dupl + distal dupl” was found in 13 individuals, all of them within haplogroup J-M304. The frequency of these two patterns has not been described previously. The same is true for the remaining overrepresented variants except for the gr/gr del. As in our data set, this variant was previously found significantly enriched in haplogroup D-M174 [48, 59].

Unfortunately, due to relatively low amount of informative Y-SNPs on Affymetrix 6.0 array, we limited our phylogenetic analysis to the main haplogroups only. In this case, it´s difficult to make reliable inferences about mutation rates of CNVs on the Y chromosome. Nevertheless, we hope that our observations will work as an implication of which variants might be common within haplogroups included in this study.

Although, the validity of the detected CNVs in the extended data set is not as precise as in the Norwegian population samples, the observation that the same CNV patterns (with minor start and stop region variations) are reoccurring in several males, strengthens the probability that these are in fact real variants (exception patterns which occur only once such as: p-arm dupl (S1A Fig), q-arm del+U3 del (S1B Fig), IR2 del (S1J Fig), Y1Y2 dupl (S1W Fig) and distal gr dupl (S1X Fig). Also, based on the concordance of the in silico detected CNVs in the Norwegian population and the results from the PCR (deletion) and qPCR (duplication) validation experiments, we are convinced that the observed CNVs in the extended data set are to large extent true. Yet we can never be 100% sure unless they all will be confirmed by molecular methods. Deciphering Developmental Disorders study (2015) has estimated the false-positive rates to about 5% and false-negative rates to <20% for technical replicates and custom designed arrays [60]. Based on these rates we estimate that the false-positive rate in our extended dataset should be approximately the similar. To decrease the rates of false discovery, we manually curated all the CNV calls which by some is regarded as “a gold standard”, we also manually inspected the remaining Y chromosomes that did not have any CNV calls. We can therefore conclude that this tedious approach helped us to keep the rate of false positive and false negative observations to an absolute minimum.

## Conclusions

For many years the analysis of the Y chromosome has been largely neglected in genome-wide studies of multiple phenotypes, because of the idea that this chromosome is poor in gene content and functionally relevant only for sex determination and testicular functions. However, renewed attention in this chromosome is due to possible association between the MSY region and multiple traits or diseases such as several types of cancer, graft versus host disease, gender differences in heart failure, sex reversal, spermatogenesis, male infertility and sex specific effects on the brain and behaviour including for example autism, and speech delay [61]. The high frequency of Y chromosome variants found in our study underlines the importance of including the Y chromosome in genome-wide studies. Not only deletions, but also duplications are of interest, since they can potentially modify the dosage of genes with important functions. Our results also show that haplogroup distribution between cases and controls has to be carefully controlled in order to avoid spurious results due to population stratification. To our knowledge, only one genome-wide study up to date has included the MSY region. This study evaluates the contribution of autosomal and both sex chromosomes in human spermatogenic failure [62]. Although they included large amounts of cases and controls from several populations, only a subset of them were analysed by Affymetrix 6.0 array, while the rest of the samples were hybridized to Illumina 370K or Illumina OmiExpress arrays, none of which contains good coverage of the MSY region (1412 or 1409 probes respectively). Interestingly, even when only a reduced population analysed with Affymetrix arrays was considered, and only individuals of R-M207 haplogroup were included, they detected higher abundance of rare duplications in cases than controls. The specific type of duplication(s) in cases and controls was not discussed in detail. Future genome-wide association studies using SNP/CN platforms with good coverage of Y chromosome have the potential to find the possible functional significance of Y chromosome variants for different phenotypes. Recent efforts have been done to generate arrays with high coverage specifically in the Y chromosome [63, 64]. While these arrays will most certainly have impact for Y chromosome related studies, including fertility, genetic anthropology and population genetics, our results shows that even the SNP/CN platforms currently used for genome-wide CNV studies would benefit from the inclusion of the Y chromosome in the analysis.

## Supporting Information

S1 Fig Signal intensity plot (Log 2 ratio) for the complete Y chromosome of 24 males. Each male contained one (or more) of the 24 different types of CNVs discovered in this study. Therefore, the figure includes one example of each type of CNV. Signals from each of the 8179 probes are represented by one dot. Signals included in regions of the Y chromosome containing ampliconic and palindromic sequences are represented by color codes, and each variant was named using the nomenclature described in Repping et al. [41] when possible, or assigned a new name when the variant was not previously described. The name of the CEL file from NCBI GEO Datasets or other sources (see Methods), containing raw data for each individual, is indicated after the name of each CNV. Staples delineate the positions of duplications or deletions.
(DOCX)

S2 Fig Dendrogram of Y chromosome haplogroups including amount of individuals assigned to each group. The amount of individuals in each group is indicated below the name of each haplogroup or internal node. The Affymetrix 6.0 arrays did not contain diagnostic SNP information for all haplogroups and three groups of individuals were assigned to internal nodes F-M89 excluding branch F1329, KLT-M9 excluding M526 and NO-M214 excluding M175. These groups, and the number of individuals in each, are listed in the top right of the Figure. Three additional groups of individuals were assigned to internal nodes DE-M145, P-P295 and IJ-M429, even when the arrays contain additional SNPs for determination of haplogroups connected to these nodes. Since not enough SNP information was available for these particular individuals, they were removed from additional analysis.
(TIF)

S3 Fig Distribution of significantly overrepresented CNVs in several haplogroups each. Each part of the Fig shows the graphical representation of the first two eigenvectors after PCA analysis. **A**: The Fig shows PCA values for individuals with P5 duplications significantly overrepresented in haplogroups E-M96, G-M201 and QM242. **B**: Individuals with P4 duplications significantly overrepresented in haplogroups KLT-M9(xM526) and Q-M242. **C**: Individuals with b1/b3 deletions (c2) significantly overrepresented in haplogroups C-M130 and O-M175.
(TIF)

S1 Table All datasets included in the study and CNV frequencies within each dataset. The table shows all datasets that are included in this study. All male samples available as well as the samples that could be assigned to a HG and included into the total set of 1506 individuals are displayed in the two first columns. Number of deletions, duplications and both deletion and duplication patterns are displayed for included samples and the all samples together with the corresponding percentage frequencies. Last column displays the overall percentages of CNVs for each study, given for both included and all samples.
(DOCX)

S2 Table SNP/CN probes in MSY exhibiting high intensity values based on hybridization of 61 female samples. The table includes the names, chromosome position and the average intensity values generated by surveillance of 61 females for SNP/CN probes with high intensity values in females compared to males. Log2R average values for three consecutive probes are also shown, since this is the minimum amount of probes that can be used in CNV detection by the Affymetrix Genotyping Console Software 4.1.3.840 (GTC). Three regions exhibited high average values (above -0.51) rendering them unsuitable for CNV detection in males.
(DOCX)

S3 Table Distribution of CNV patterns and their frequency within haplogroups. The table shows the distribution of CNV patterns among haplogroups for 15 variants that did not show overrepresentation in any group. Ambiguous individuals, for which haplotype determination was not possible, are shown in the table for completeness, but they were not included in the statistical analysis. Two individuals exhibited CNV patterns that were not possible to classify into any variant category, and they are therefore listed as “unspecified”.
(DOCX)

## References

[1] Riggs ER, Ledbetter DH, Martin CL. Genomic Variation: Lessons Learned from Whole-Genome CNV Analysis. Current genetic medicine reports. 2014;2:146–50. 10.1007/s40142-014-0048-4 25152847 PMC4129219

[2] Aston KI. Genetic susceptibility to male infertility: news from genome-wide association studies. Andrology. 2014;2(3):315–21. Epub 2014/02/28. 10.1111/j.2047-2927.2014.00188.x . 24574159

[3] Lu C, Zhang F, Yang H, Xu M, Du G, Wu W, et al Additional genomic duplications in AZFc underlie the b2/b3 deletion-associated risk of spermatogenic impairment in Han Chinese population. Human molecular genetics. 2011;20(22):4411–21. 10.1093/hmg/ddr369 . 21852246

[4] Forsberg LA, Rasi C, Malmqvist N, Davies H, Pasupulati S, Pakalapati G, et al Mosaic loss of chromosome Y in peripheral blood is associated with shorter survival and higher risk of cancer. Nature genetics. 2014;46(6):624–8. 10.1038/ng.2966 . 24777449 PMC5536222

[5] Dumanski JP, Rasi C, Lonn M, Davies H, Ingelsson M, Giedraitis V, et al Mutagenesis. Smoking is associated with mosaic loss of chromosome Y. Science. 2015;347(6217):81–3. 10.1126/science.1262092 . 25477213 PMC4356728

[6] King TE, Jobling MA. What's in a name? Y chromosomes, surnames and the genetic genealogy revolution. Trends in genetics: TIG. 2009;25(8):351–60. Epub 2009/08/12. 10.1016/j.tig.2009.06.003 . 19665817

[7] Larmuseau MH, Vanoverbeke J, Van Geystelen A, Defraene G, Vanderheyden N, Matthys K, et al Low historical rates of cuckoldry in a Western European human population traced by Y-chromosome and genealogical data. Proceedings Biological sciences / The Royal Society. 2013;280(1772):20132400 24266034 10.1098/rspb.2013.2400 PMC3813347

[8] Tozzo P, Giuliodori A, Corato S, Ponzano E, Rodriguez D, Caenazzo L. Deletion of amelogenin Y-locus in forensics: literature revision and description of a novel method for sex confirmation. Journal of forensic and legal medicine. 2013;20(5):387–91. Epub 2013/06/13. 10.1016/j.jflm.2013.03.012 . 23756502

[9] Repnikova EA, Rosenfeld JA, Bailes A, Weber C, Erdman L, McKinney A, et al Characterization of copy number variation in genomic regions containing STR loci using array comparative genomic hybridization. Forensic science international Genetics. 2013;7(5):475–81. Epub 2013/08/21. 10.1016/j.fsigen.2013.05.008 . 23948316

[10] Hallast P, Batini C, Zadik D, Maisano Delser P, Wetton JH, Arroyo-Pardo E, et al The Y-Chromosome Tree Bursts into Leaf: 13,000 High-Confidence SNPs Covering the Majority of Known Clades. Mol Biol Evol. 2014 Epub 2014/12/04. 10.1093/molbev/msu327 . 25468874 PMC4327154

[11] Larmuseau MH, Ottoni C, Raeymaekers JA, Vanderheyden N, Larmuseau HF, Decorte R. Temporal differentiation across a West-European Y-chromosomal cline: genealogy as a tool in human population genetics. European journal of human genetics: EJHG. 2012;20(4):434–40. Epub 2011/12/01. 10.1038/ejhg.2011.218 ; PubMed Central PMCID: PMCPmc3306861. 22126748 PMC3306861

[12] Jamain S, Quach H, Quintana-Murci L, Betancur C, Philippe A, Gillberg C, et al Y chromosome haplogroups in autistic subjects. Molecular psychiatry. 2002;7(2):217–9. 10.1038/sj.mp.4000968 11840316 PMC1899172

[13] McCarthy MM, Arnold AP. Reframing sexual differentiation of the brain. Nature neuroscience. 2011;14(6):677–83. Epub 2011/05/27. 10.1038/nn.2834 ; PubMed Central PMCID: PMCPmc3165173. 21613996 PMC3165173

[14] Talseth-Palmer BA, Holliday EG, Evans TJ, McEvoy M, Attia J, Grice DM, et al Continuing difficulties in interpreting CNV data: lessons from a genome-wide CNV association study of Australian HNPCC/lynch syndrome patients. BMC medical genomics. 2013;6:10 Epub 2013/03/28. 10.1186/1755-8794-6-10 ; PubMed Central PMCID: PMCPmc3626775. 23531357 PMC3626775

[15] Hughes JF, Skaletsky H, Pyntikova T, Graves TA, van Daalen SK, Minx PJ, et al Chimpanzee and human Y chromosomes are remarkably divergent in structure and gene content. Nature. 2010;463(7280):536–9. 10.1038/nature08700 20072128 PMC3653425

[16] Kuroda-Kawaguchi T, Skaletsky H, Brown LG, Minx PJ, Cordum HS, Waterston RH, et al The AZFc region of the Y chromosome features massive palindromes and uniform recurrent deletions in infertile men. Nature genetics. 2001;29(3):279–86. 10.1038/ng757 . 11687796

[17] Skaletsky H, Kuroda-Kawaguchi T, Minx PJ, Cordum HS, Hillier L, Brown LG, et al The male-specific region of the human Y chromosome is a mosaic of discrete sequence classes. Nature. 2003;423(6942):825–37. 10.1038/nature01722 . 12815422

[18] Kozina V, Cappallo-Obermann H, Gromoll J, Spiess AN. A one-step real-time multiplex PCR for screening Y-chromosomal microdeletions without downstream amplicon size analysis. PloS one. 2011;6(8):e23174 10.1371/journal.pone.0023174 21887237 PMC3161745

[19] Qureshi SJ, Ross AR, Ma K, Cooke HJ, Intyre MA, Chandley AC, et al Polymerase chain reaction screening for Y chromosome microdeletions: a first step towards the diagnosis of genetically-determined spermatogenic failure in men. Molecular human reproduction. 1996;2(10):775–9. Epub 1996/10/01. . 9239696 10.1093/molehr/2.10.775

[20] Mancini TI, Oliveira MM, Dutra AR, Perez AB, Minillo RM, Takeno SS, et al Interstitial 4q Deletion and Isodicentric Y-Chromosome in a Patient with Dysmorphic Features. Molecular syndromology. 2012;3(1):39–43. Epub 2012/08/03. doi: 000338468. ; PubMed Central PMCID: PMCPmc3398833. 22855654 10.1159/000338468 PMC3398833

[21] Lange J, Skaletsky H, van Daalen SK, Embry SL, Korver CM, Brown LG, et al Isodicentric Y chromosomes and sex disorders as byproducts of homologous recombination that maintains palindromes. Cell. 2009;138(5):855–69. 10.1016/j.cell.2009.07.042 19737515 PMC3616640

[22] Martinez-Moczygemba M, Doan ML, Elidemir O, Fan LL, Cheung SW, Lei JT, et al Pulmonary alveolar proteinosis caused by deletion of the GM-CSFRalpha gene in the X chromosome pseudoautosomal region 1. The Journal of experimental medicine. 2008;205(12):2711–6. Epub 2008/10/29. 10.1084/jem.20080759 ; PubMed Central PMCID: PMCPmc2585851. 18955567 PMC2585851

[23] Helena Mangs A, Morris BJ. The Human Pseudoautosomal Region (PAR): Origin, Function and Future. Current genomics. 2007;8(2):129–36. Epub 2008/07/29. ; PubMed Central PMCID: PMCPmc2435358. 18660847 10.2174/138920207780368141 PMC2435358

[24] Jorgez CJ, Weedin JW, Sahin A, Tannour-Louet M, Han S, Bournat JC, et al Aberrations in pseudoautosomal regions (PARs) found in infertile men with Y-chromosome microdeletions. The Journal of clinical endocrinology and metabolism. 2011;96(4):E674–9. Epub 2011/01/22. 10.1210/jc.2010-2018 ; PubMed Central PMCID: PMCPmc3070254. 21252244 PMC3070254

[25] Mensah MA, Hestand MS, Larmuseau MH, Isrie M, Vanderheyden N, Declercq M, et al Pseudoautosomal region 1 length polymorphism in the human population. PLoS genetics. 2014;10(11):e1004578 10.1371/journal.pgen.1004578 25375121 PMC4222609

[26] Djurovic S, Gustafsson O, Mattingsdal M, Athanasiu L, Bjella T, Tesli M, et al A genome-wide association study of bipolar disorder in Norwegian individuals, followed by replication in Icelandic sample. Journal of affective disorders. 2010;126(1–2):312–6. Epub 2010/05/11. 10.1016/j.jad.2010.04.007 . 20451256

[27] Athanasiu L, Mattingsdal M, Kahler AK, Brown A, Gustafsson O, Agartz I, et al Gene variants associated with schizophrenia in a Norwegian genome-wide study are replicated in a large European cohort. Journal of psychiatric research. 2010;44(12):748–53. 10.1016/j.jpsychires.2010.02.002 20185149 PMC3224994

[28] Rimol LM, Hartberg CB, Nesvag R, Fennema-Notestine C, Hagler DJ Jr, Pung CJ, et al Cortical thickness and subcortical volumes in schizophrenia and bipolar disorder. Biological psychiatry. 2010;68(1):41–50. Epub 2010/07/09. 10.1016/j.biopsych.2010.03.036 . 20609836

[29] Edgar R, Domrachev M, Lash AE. Gene Expression Omnibus: NCBI gene expression and hybridization array data repository. Nucleic acids research. 2002;30(1):207–10. Epub 2001/12/26. ; PubMed Central PMCID: PMCPmc99122. 11752295 10.1093/nar/30.1.207 PMC99122

[30] Simonson TS, Yang Y, Huff CD, Yun H, Qin G, Witherspoon DJ, et al Genetic evidence for high-altitude adaptation in Tibet. Science. 2010;329(5987):72–5. 10.1126/science.1189406 . 20466884

[31] Watkins WS, Xing J, Huff C, Witherspoon DJ, Zhang Y, Perego UA, et al Genetic analysis of ancestry, admixture and selection in Bolivian and Totonac populations of the New World. BMC genetics. 2012;13:39 10.1186/1471-2156-13-39 22606979 PMC3432609

[32] Lou H, Li S, Yang Y, Kang L, Zhang X, Jin W, et al A map of copy number variations in Chinese populations. PloS one. 2011;6(11):e27341 10.1371/journal.pone.0027341 22087296 PMC3210162

[33] Mao X, Yu Y, Boyd LK, Ren G, Lin D, Chaplin T, et al Distinct genomic alterations in prostate cancers in Chinese and Western populations suggest alternative pathways of prostate carcinogenesis. Cancer research. 2010;70(13):5207–12. 10.1158/0008-5472.CAN-09-4074 20516122 PMC2896548

[34] Mulle JG, Dodd AF, McGrath JA, Wolyniec PS, Mitchell AA, Shetty AC, et al Microdeletions of 3q29 confer high risk for schizophrenia. American journal of human genetics. 2010;87(2):229–36. 10.1016/j.ajhg.2010.07.013 20691406 PMC2917706

[35] Bray SM, Mulle JG, Dodd AF, Pulver AE, Wooding S, Warren ST. Signatures of founder effects, admixture, and selection in the Ashkenazi Jewish population. Proceedings of the National Academy of Sciences of the United States of America. 2010;107(37):16222–7. 10.1073/pnas.1004381107 20798349 PMC2941333

[36] Venkatachalam R, Verwiel ET, Kamping EJ, Hoenselaar E, Gorgens H, Schackert HK, et al Identification of candidate predisposing copy number variants in familial and early-onset colorectal cancer patients. International journal of cancer Journal international du cancer. 2011;129(7):1635–42. Epub 2010/12/04. 10.1002/ijc.25821 . 21128281

[37] The International HapMap Project. Nature. 2003;426(6968):789–96. Epub 2003/12/20. 10.1038/nature02168 . 14685227

[38] Siggberg L, Sirpa AM, Tarja L, Kristiina A, Ilari S, Kati K, et al High-resolution SNP array analysis of patients with developmental disorder and normal array CGH results. BMC medical genetics. 2012;13:84 10.1186/1471-2350-13-84 22984989 PMC3523000

[39] Ferlin A, Tessari A, Ganz F, Marchina E, Barlati S, Garolla A, et al Association of partial AZFc region deletions with spermatogenic impairment and male infertility. Journal of medical genetics. 2005;42(3):209–13. 10.1136/jmg.2004.025833 15744033 PMC1736009

[40] Hallast P, Balaresque P, Bowden GR, Ballereau S, Jobling MA. Recombination dynamics of a human Y-chromosomal palindrome: rapid GC-biased gene conversion, multi-kilobase conversion tracts, and rare inversions. PLoS genetics. 2013;9(7):e1003666 10.1371/journal.pgen.1003666 23935520 PMC3723533

[41] Repping S, van Daalen SK, Brown LG, Korver CM, Lange J, Marszalek JD, et al High mutation rates have driven extensive structural polymorphism among human Y chromosomes. Nature genetics. 2006;38(4):463–7. 10.1038/ng1754 . 16501575

[42] Ferlin A, Arredi B, Speltra E, Cazzadore C, Selice R, Garolla A, et al Molecular and clinical characterization of Y chromosome microdeletions in infertile men: a 10-year experience in Italy. The Journal of clinical endocrinology and metabolism. 2007;92(3):762–70. 10.1210/jc.2006-1981 . 17213277

[43] Van Geystelen A, Wenseleers T, Decorte R, Caspers MJ, Larmuseau MH. In silico detection of phylogenetic informative Y-chromosomal single nucleotide polymorphisms from whole genome sequencing data. Electrophoresis. 2014;35(21–22):3102–10. 10.1002/elps.201300459 . 24615884

[44] Van Geystelen A, Decorte R, Larmuseau MH. AMY-tree: an algorithm to use whole genome SNP calling for Y chromosomal phylogenetic applications. BMC genomics. 2013;14:101 10.1186/1471-2164-14-101 23405914 PMC3583733

[45] Van Geystelen A, Decorte R, Larmuseau MH. Updating the Y-chromosomal phylogenetic tree for forensic applications based on whole genome SNPs. Forensic science international Genetics. 2013;7(6):573–80. Epub 2013/04/20. 10.1016/j.fsigen.2013.03.010 . 23597787

[46] Repping S, van Daalen SK, Korver CM, Brown LG, Marszalek JD, Gianotten J, et al A family of human Y chromosomes has dispersed throughout northern Eurasia despite a 1.8-Mb deletion in the azoospermia factor c region. Genomics. 2004;83(6):1046–52. 10.1016/j.ygeno.2003.12.018 . 15177557

[47] Lin YW, Thi DA, Kuo PL, Hsu CC, Huang BD, Yu YH, et al Polymorphisms associated with the DAZ genes on the human Y chromosome. Genomics. 2005;86(4):431–8. Epub 2005/08/09. 10.1016/j.ygeno.2005.07.003 . 16085382

[48] Repping S, Skaletsky H, Brown L, van Daalen SK, Korver CM, Pyntikova T, et al Polymorphism for a 1.6-Mb deletion of the human Y chromosome persists through balance between recurrent mutation and haploid selection. Nature genetics. 2003;35(3):247–51. 10.1038/ng1250 . 14528305

[49] Plaseski T, Noveski P, Trivodalieva S, Efremov GD, Plaseska-Karanfilska D. Quantitative fluorescent-PCR detection of sex chromosome aneuploidies and AZF deletions/duplications. Genetic testing. 2008;12(4):595–605. Epub 2008/12/17. 10.1089/gte.2008.0068 . 19072570

[50] Krausz C, Giachini C, Forti G. TSPY and Male Fertility. Genes. 2010;1(2):308–16. 10.3390/genes1020308 24710048 PMC3954084

[51] Kido T, Hatakeyama S, Ohyama C, Lau YF. Expression of the Y-Encoded TSPY is Associated with Progression of Prostate Cancer. Genes. 2010;1(2):283–93. Epub 2010/01/01. 10.3390/genes1020283 ; PubMed Central PMCID: PMCPmc3954091. 24710046 PMC3954091

[52] Rozen SG, Marszalek JD, Irenze K, Skaletsky H, Brown LG, Oates RD, et al AZFc deletions and spermatogenic failure: a population-based survey of 20,000 Y chromosomes. American journal of human genetics. 2012;91(5):890–6. 10.1016/j.ajhg.2012.09.003 23103232 PMC3487143

[53] Redon R, Ishikawa S, Fitch KR, Feuk L, Perry GH, Andrews TD, et al Global variation in copy number in the human genome. Nature. 2006;444(7118):444–54. Epub 2006/11/24. 10.1038/nature05329 ; PubMed Central PMCID: PMCPmc2669898. 17122850 PMC2669898

[54] Jobling MA. Copy number variation on the human Y chromosome. Cytogenetic and genome research. 2008;123(1–4):253–62. 10.1159/000184715 . 19287162

[55] Fietz SA, Lachmann R, Brandl H, Kircher M, Samusik N, Schroder R, et al Transcriptomes of germinal zones of human and mouse fetal neocortex suggest a role of extracellular matrix in progenitor self-renewal. Proceedings of the National Academy of Sciences of the United States of America. 2012;109(29):11836–41. Epub 2012/07/04. 10.1073/pnas.1209647109 ; PubMed Central PMCID: PMCPmc3406833. 22753484 PMC3406833

[56] Betran E, Demuth JP, Williford A. Why chromosome palindromes? International journal of evolutionary biology. 2012;2012:207958 10.1155/2012/207958 22844637 PMC3403216

[57] Conrad DF, Pinto D, Redon R, Feuk L, Gokcumen O, Zhang Y, et al Origins and functional impact of copy number variation in the human genome. Nature. 2010;464(7289):704–12. 10.1038/nature08516 19812545 PMC3330748

[58] van Oven M, Van Geystelen A, Kayser M, Decorte R, Larmuseau MH. Seeing the wood for the trees: a minimal reference phylogeny for the human Y chromosome. Human mutation. 2014;35(2):187–91. 10.1002/humu.22468 . 24166809

[59] Sin HS, Koh E, Shigehara K, Sugimoto K, Maeda Y, Yoshida A, et al Features of constitutive gr/gr deletion in a Japanese population. Human reproduction. 2010;25(9):2396–403. Epub 2010/07/29. 10.1093/humrep/deq191 . 20663794

[60] Large-scale discovery of novel genetic causes of developmental disorders. Nature. 2015;519(7542):223–8. Epub 2014/12/24. 10.1038/nature14135 . 25533962 PMC5955210

[61] Jangravi Z, Alikhani M, Arefnezhad B, Sharifi Tabar M, Taleahmad S, Karamzadeh R, et al A fresh look at the male-specific region of the human Y chromosome. Journal of proteome research. 2013;12(1):6–22. 10.1021/pr300864k . 23253012

[62] Lopes AM, Aston KI, Thompson E, Carvalho F, Goncalves J, Huang N, et al Human spermatogenic failure purges deleterious mutation load from the autosomes and both sex chromosomes, including the gene DMRT1. PLoS genetics. 2013;9(3):e1003349 10.1371/journal.pgen.1003349 23555275 PMC3605256

[63] Yuen RK, Merkoulovitch A, MacDonald JR, Vlasschaert M, Lo K, Grober E, et al Development of a high-resolution Y-chromosome microarray for improved male infertility diagnosis. Fertility and sterility. 2014;101(4):1079–85 e3. 10.1016/j.fertnstert.2013.12.027 . 24462061

[64] Elhaik E, Greenspan E, Staats S, Krahn T, Tyler-Smith C, Xue Y, et al The GenoChip: a new tool for genetic anthropology. Genome biology and evolution. 2013;5(5):1021–31. 10.1093/gbe/evt066 23666864 PMC3673633

