# Supplementary material for: Microarray Analysis of Copy Number Variants on the Human Y Chromosome Reveals Novel and Frequent Duplications Overrepresented in Specific Haplogroups
Source: PLoS One. 2015 Aug 31;10(8):e0137223. doi: 10.1371/journal.pone.0137223 (PMC4554990; doi:10.1371/journal.pone.0137223)

Supplementary Figure 1 – Representation of detected CNV patterns

1. p-arm dupl (Picul 181448)


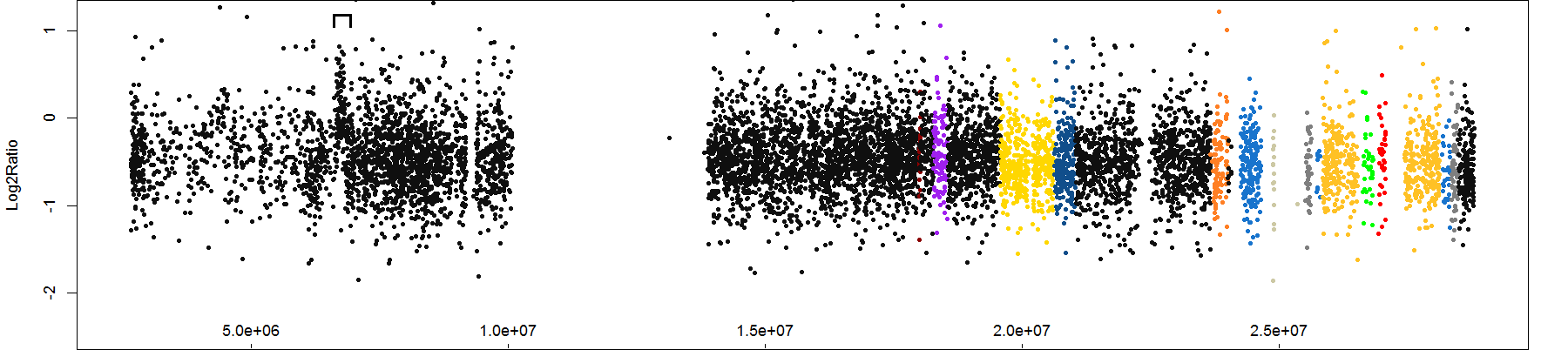


1. q-arm dupl + U3 del (Sakes 181784)


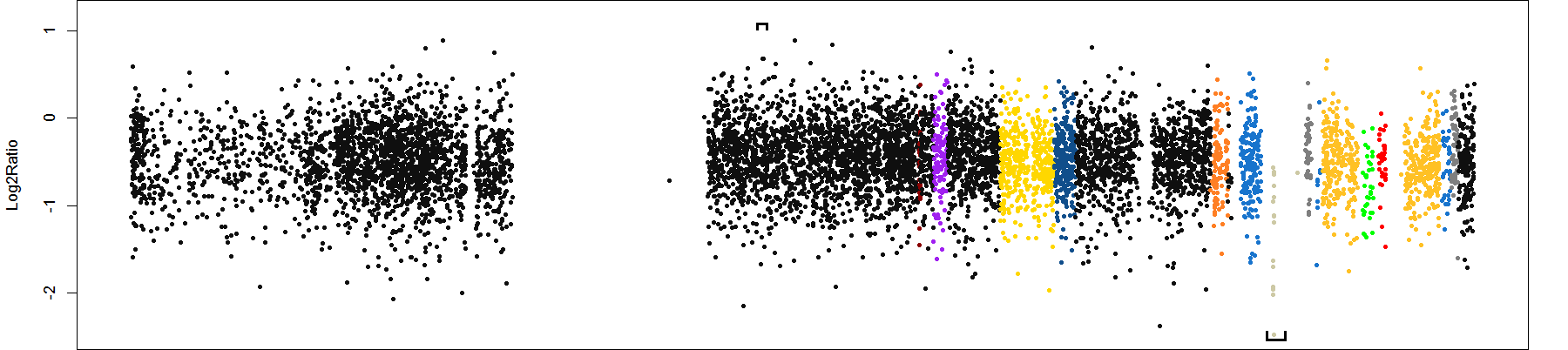


1. q-arm del (Picul 181558)


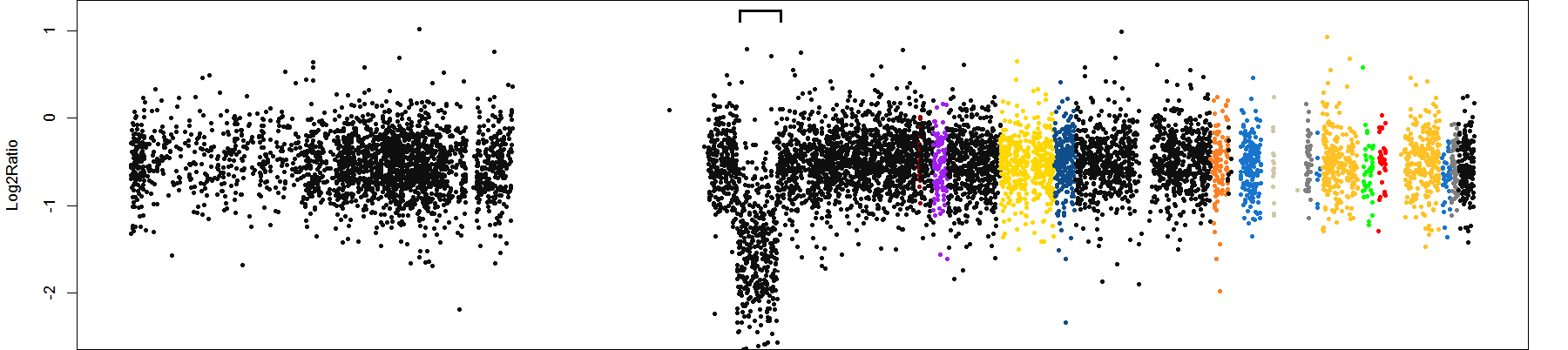


1. Palindrome 6 dupl (K5415)


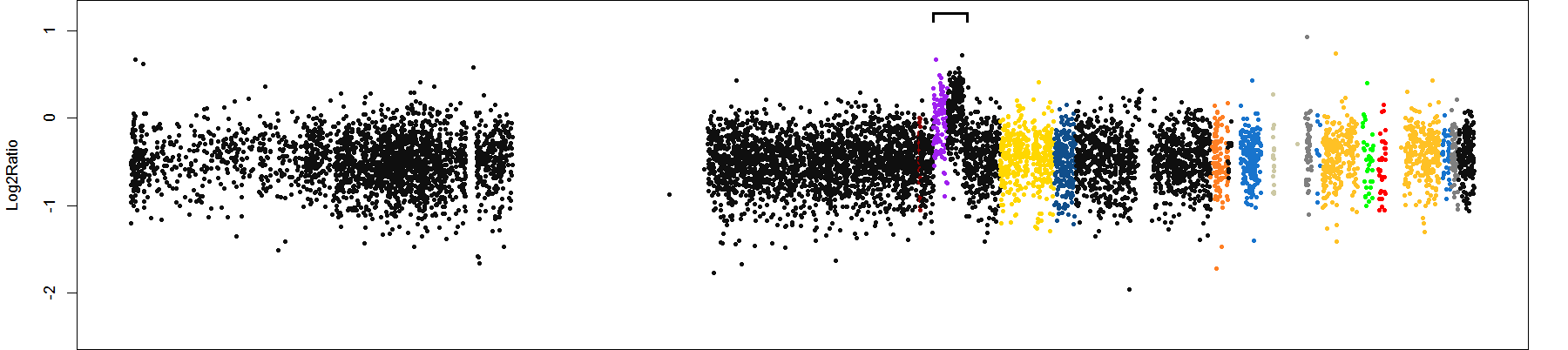


1. Palindrome 5 dupl (GSM579696)


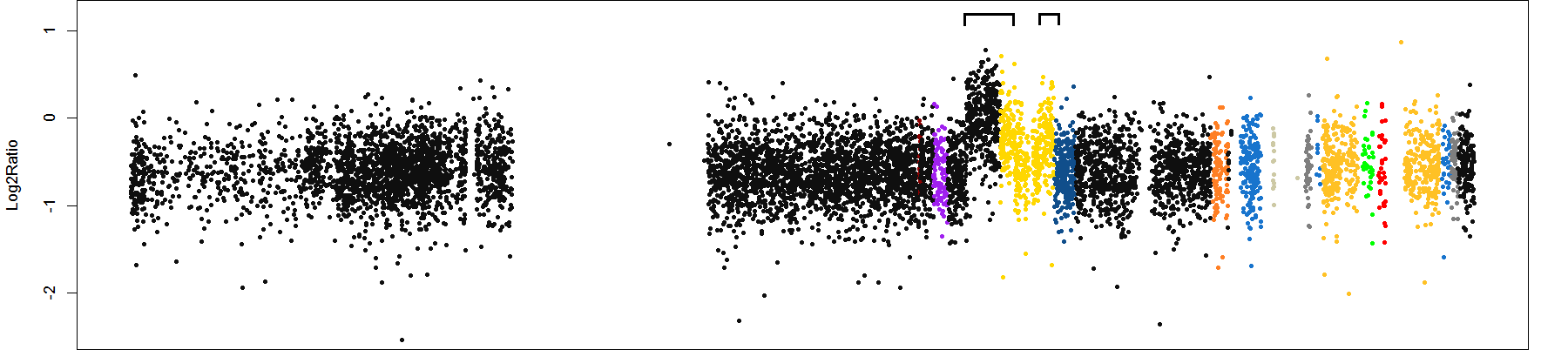


1. Prior P5 post P4 duplication (Huffs 218174)


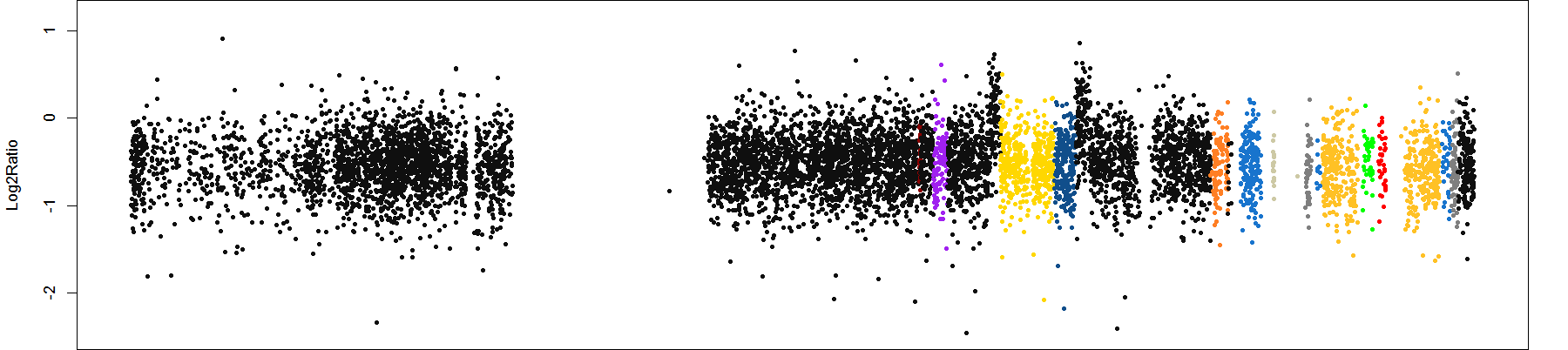


Supplementary Figure 1 (continuation)

1. Palindrome 5 del (Sloth 218404)


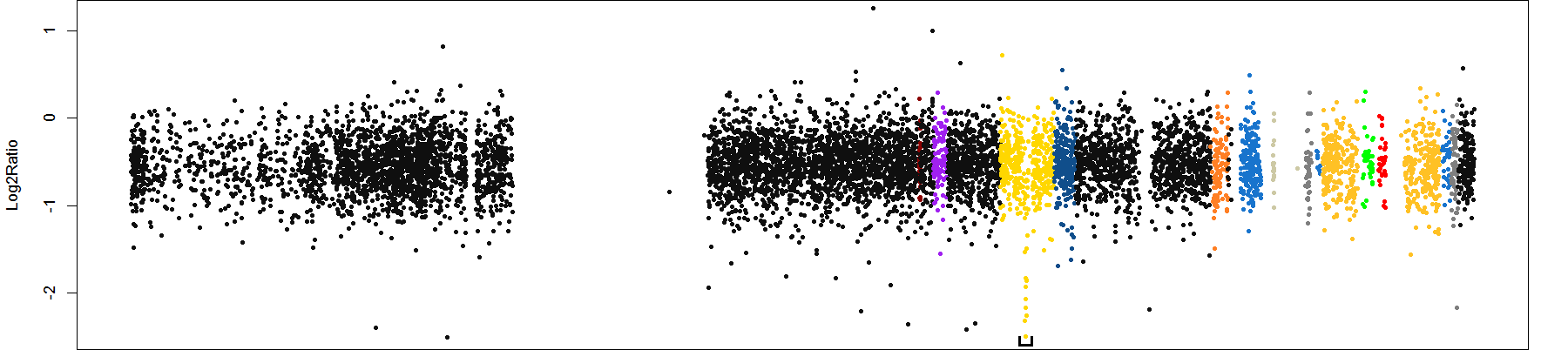


1. Palindrome 4 dupl GSM756215)


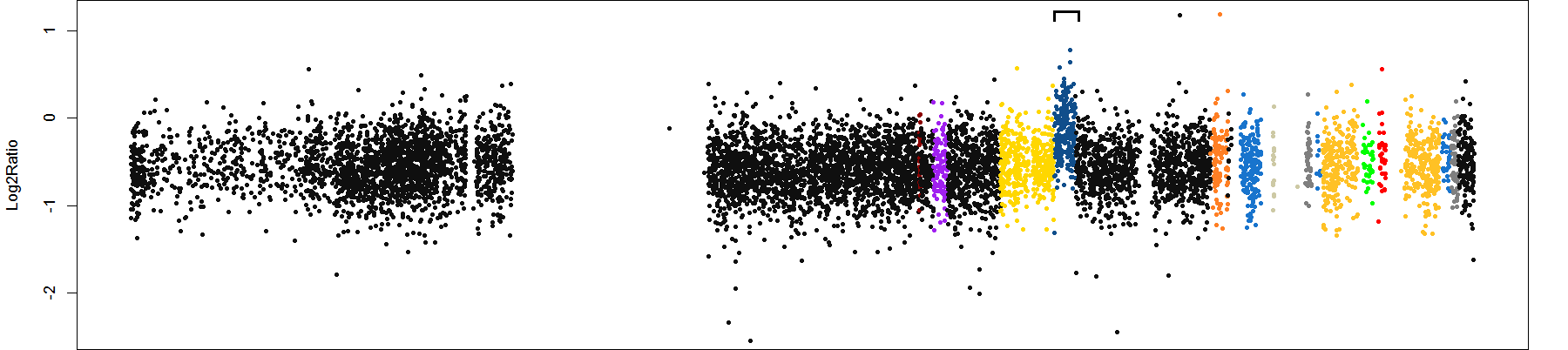


1. IR2 dupl (GSM574313)


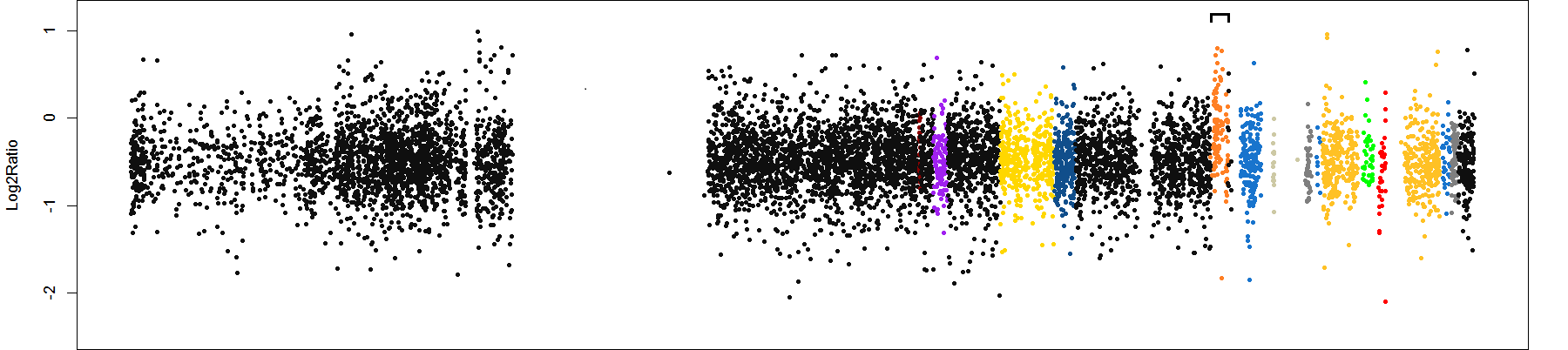


1. IR2 del (GSM457716)


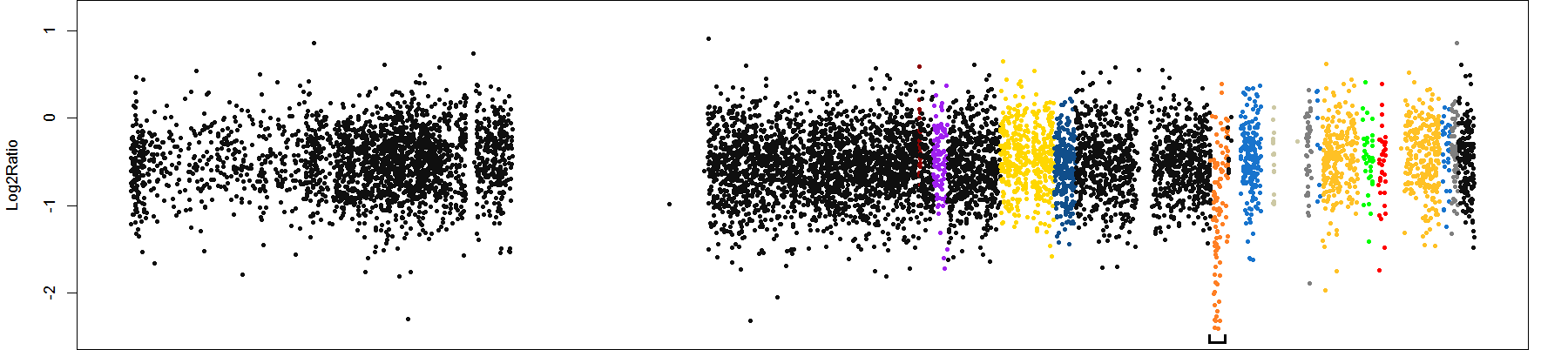


1. Palindrome 3 dupl (GSM579678)


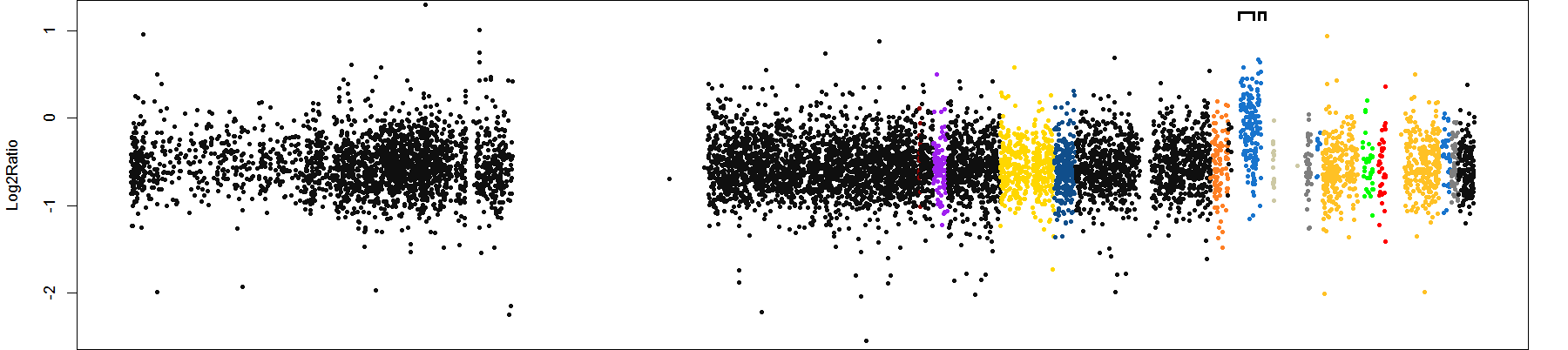


1. Palindrome 3 del (EPODE 181138)


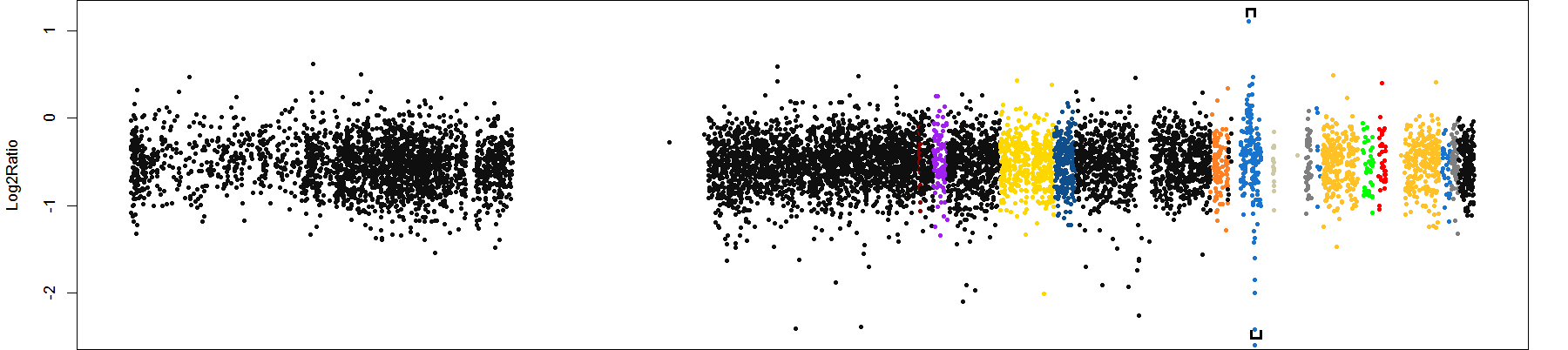


Supplementary Figure 1 (continuation)

1. b1/b3 del (GSM575067, c2)


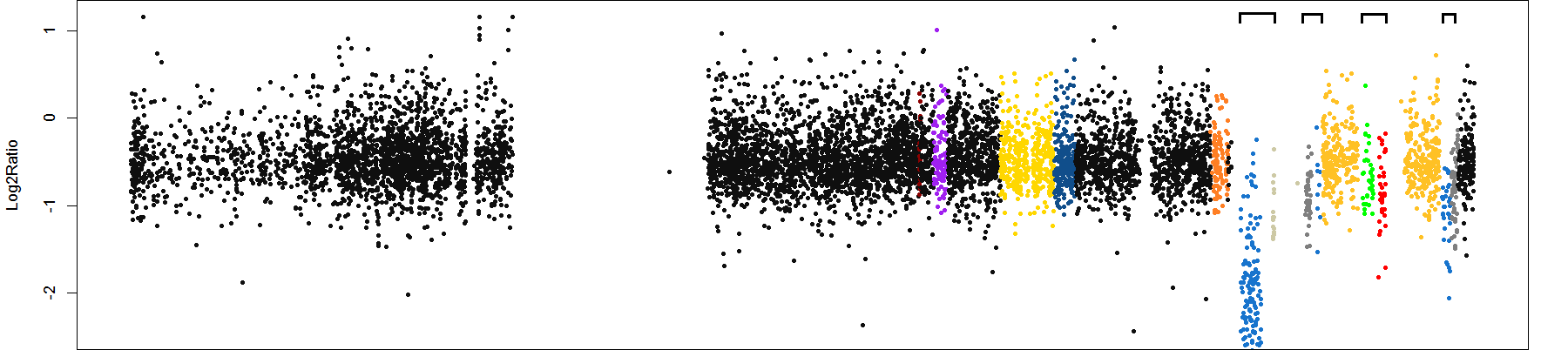


1. b2/b3 del (I7134, c35)


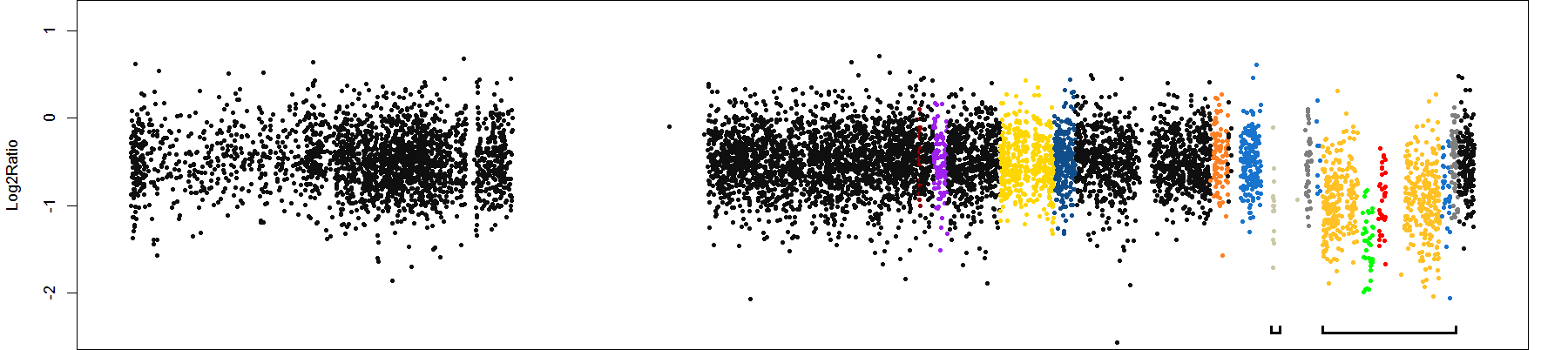


1. Blue-grey dupl (K5280, c449)


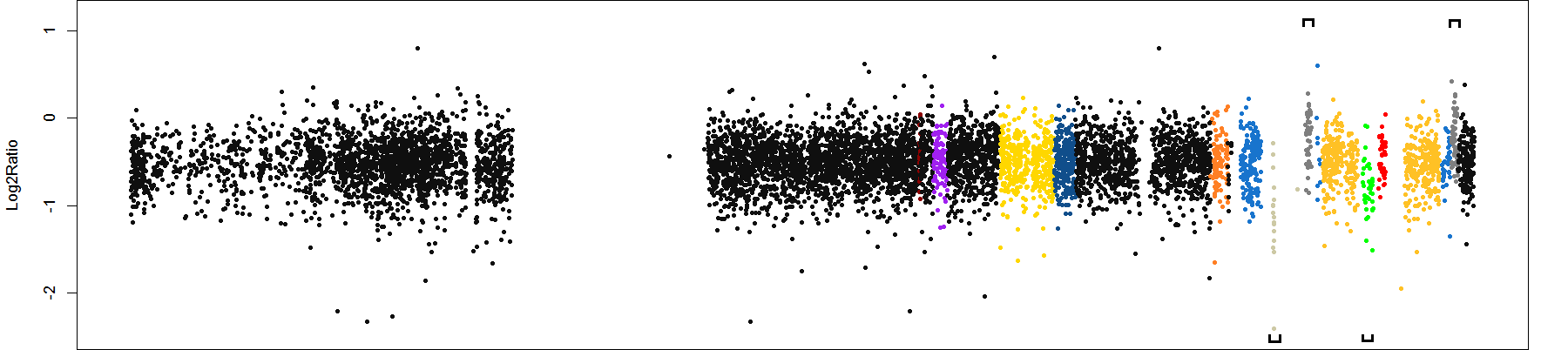


1. Blue-grey like dupl (GSM755929)


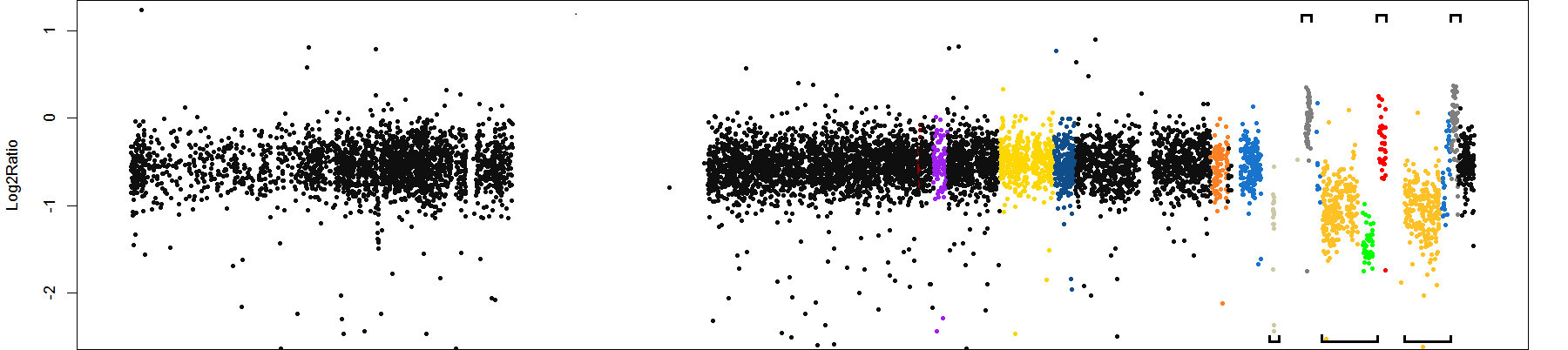


1. b2/b4 del dupl (U512, C6)


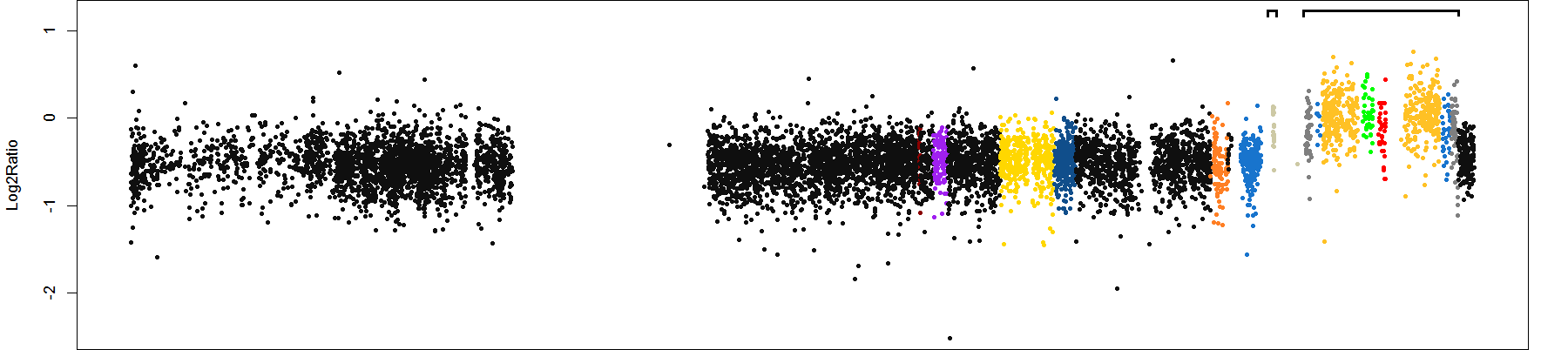


1. b2/b4 dupl (Epode 180050, c21)


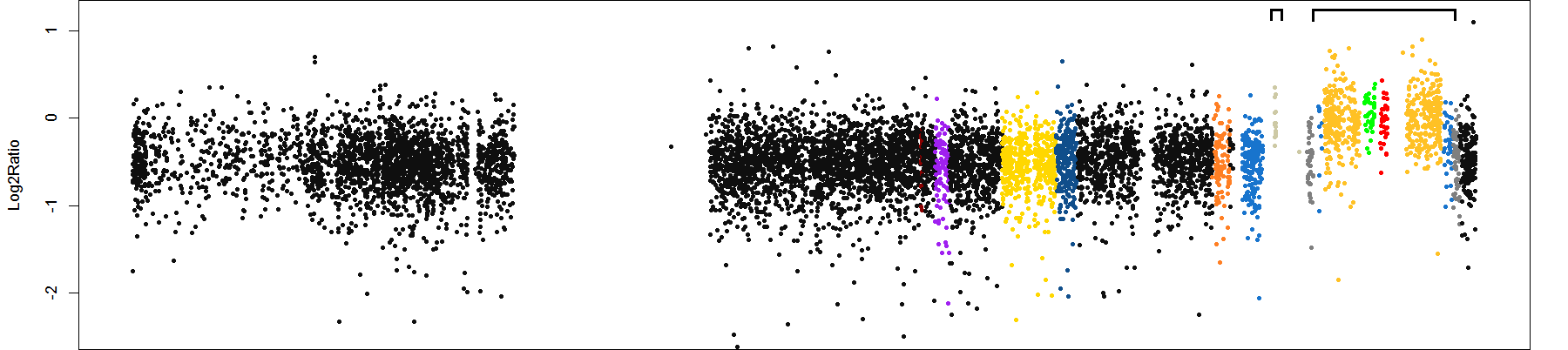


Supplementary Figure 1 (continuation)

1. b2/b4 dupl (GSM755939, c56)


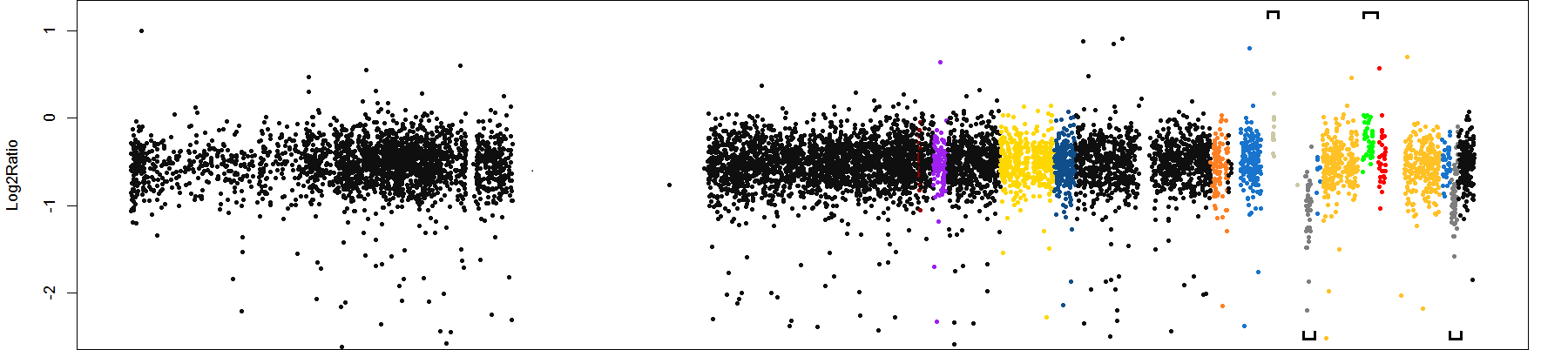


1. gr/gr dupl (K5172, c9)


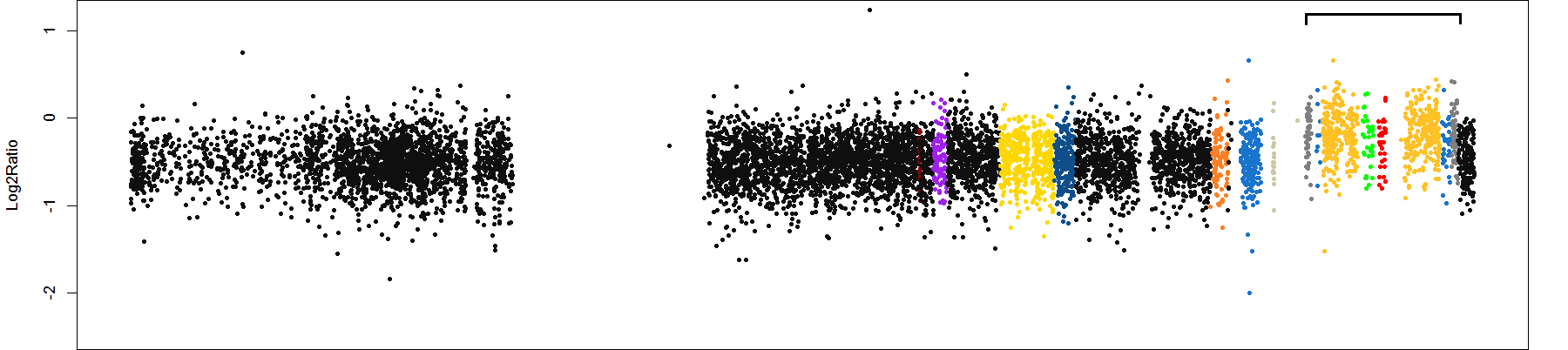


1. gr/gr dupl + distal dupl (GSM574459)


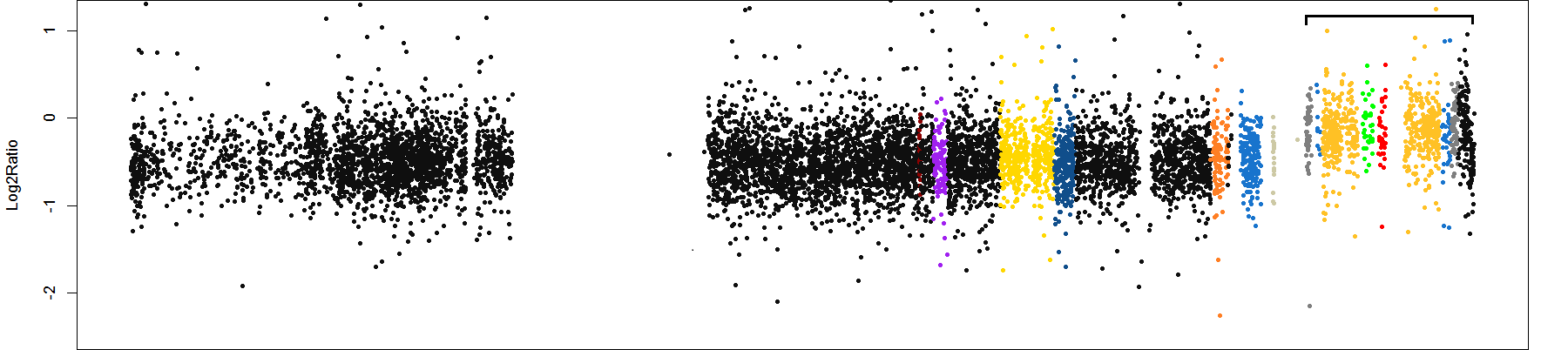


1. gr/gr del (U842, c8)


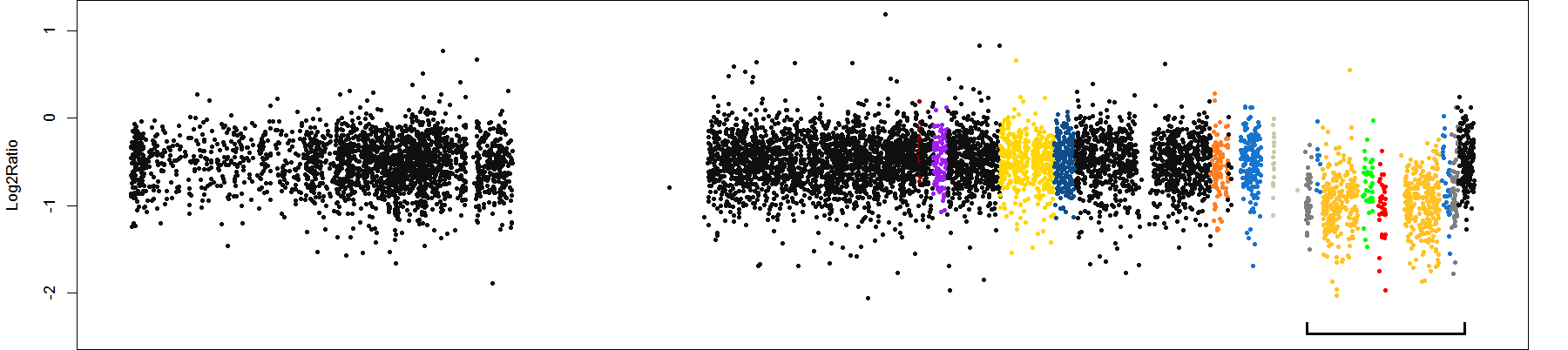


1. Y1Y2 dupl (Picul 181406)


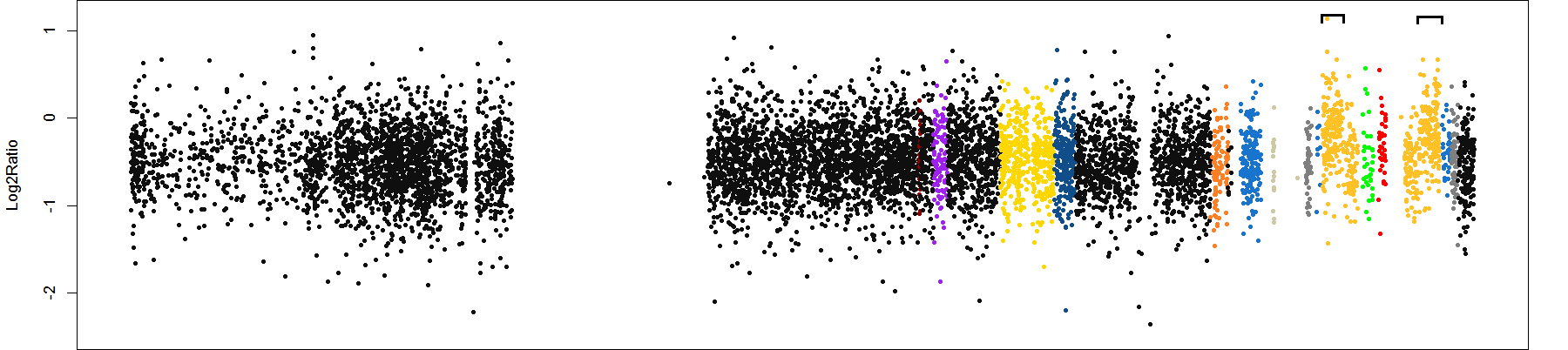


1. Distal gr dupl (Nighs 196526)


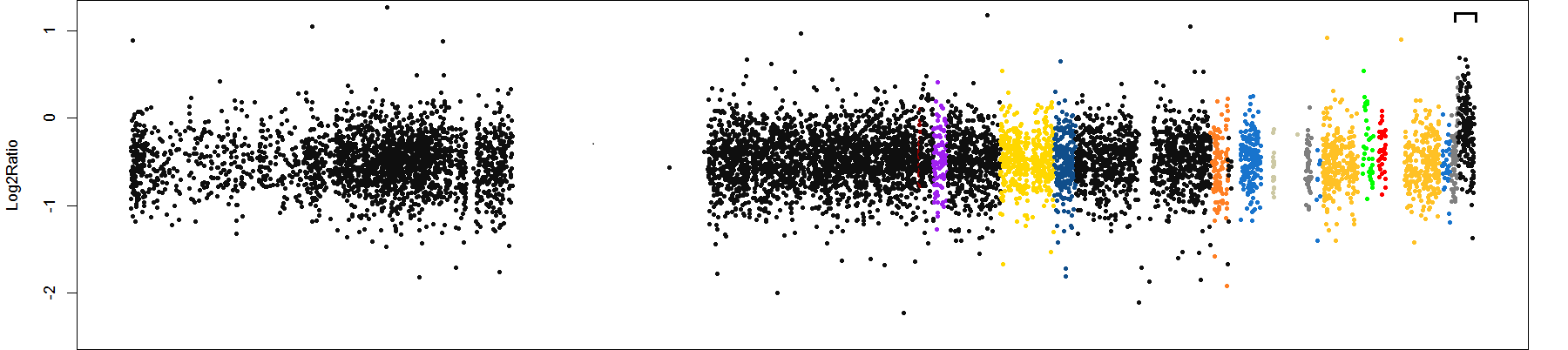


1. Distal gr del (GSM574712)


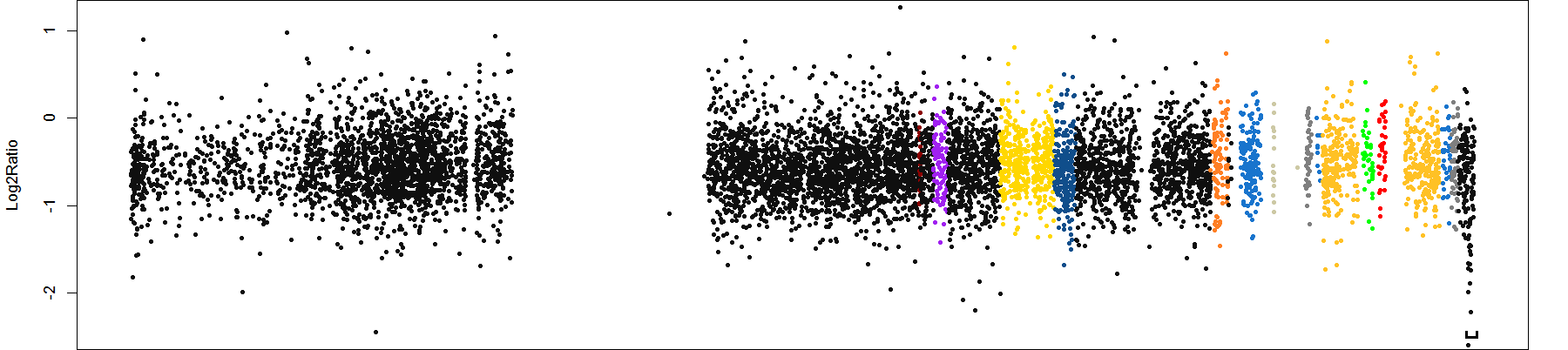

Supplement: S1 Fig — Each male contained one (or more) of the 24 different types of CNVs discovered in this study. Therefore, the figure includes one example of each type of CNV. Signals from each of the 8179 probes are represented by one dot. Signals included in regions of the Y chromosome containing ampliconic and palindromic sequences are represented by color codes, and each variant was named using the nomenclature described in Repping et al. [41] when possible, or assigned a new name when the variant was not previously described. The name of the CEL file from NCBI GEO Datasets or other sources (see Methods), containing raw data for each individual, is indicated after the name of each CNV. Staples delineate the positions of duplications or deletions. (DOCX) [file pone.0137223.s001.docx]
