# Supplementary figures and images for: Microarray Analysis of Copy Number Variants on the Human Y Chromosome Reveals Novel and Frequent Duplications Overrepresented in Specific Haplogroups
Source: PLoS One. 2015 Aug 31;10(8):e0137223. doi: 10.1371/journal.pone.0137223 (PMC4554990; doi:10.1371/journal.pone.0137223)

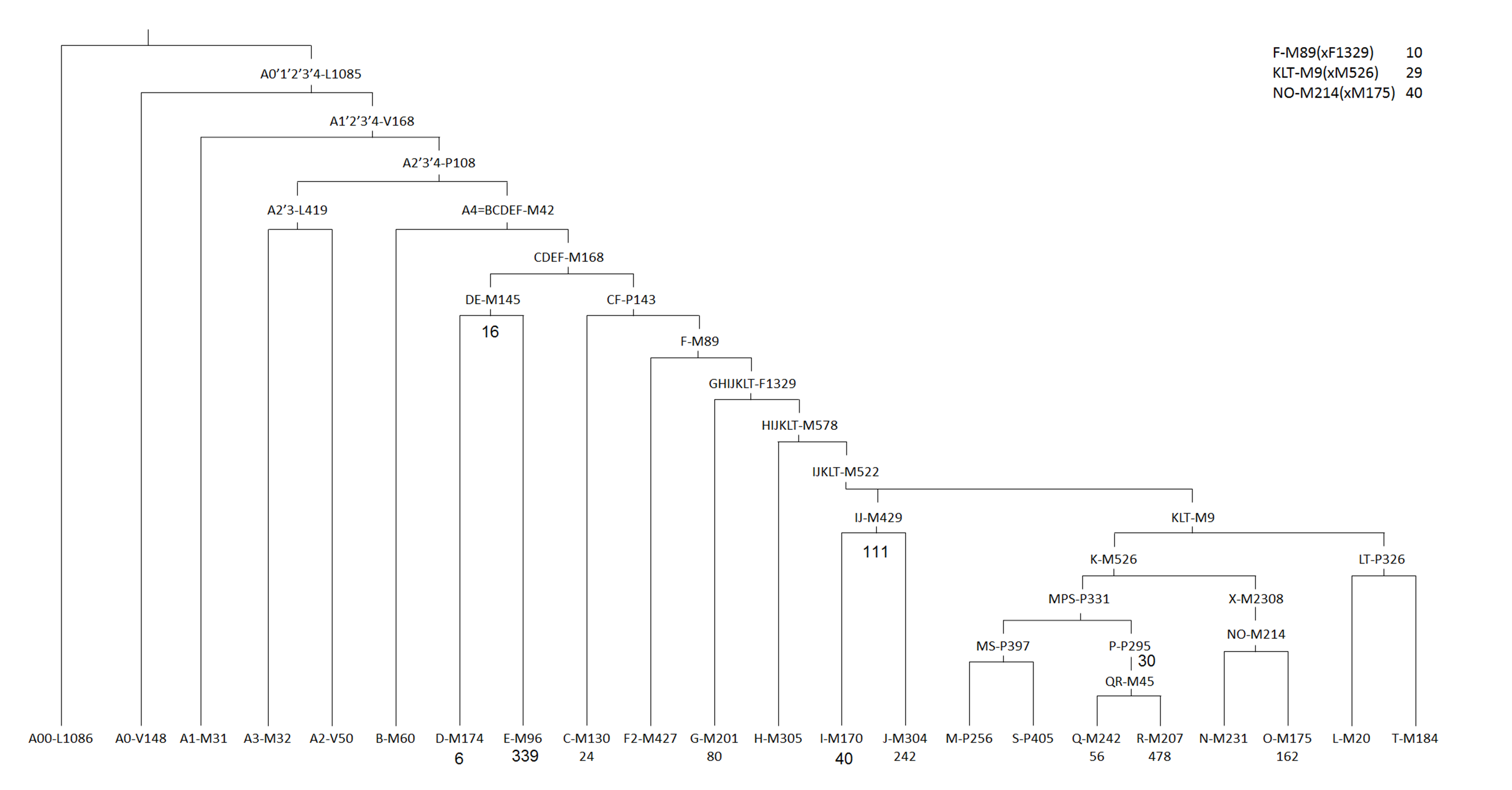

Supplement: S2 Fig — The amount of individuals in each group is indicated below the name of each haplogroup or internal node. The Affymetrix 6.0 arrays did not contain diagnostic SNP information for all haplogroups and three groups of individuals were assigned to internal nodes F-M89 excluding branch F1329, KLT-M9 excluding M526 and NO-M214 excluding M175. These groups, and the number of individuals in each, are listed in the top right of the Figure. Three additional groups of individuals were assigned to internal nodes DE-M145, P-P295 and IJ-M429, even when the arrays contain additional SNPs for determination of haplogroups connected to these nodes. Since not enough SNP information was available for these particular individuals, they were removed from additional analysis. (TIF) [file pone.0137223.s002.tif]

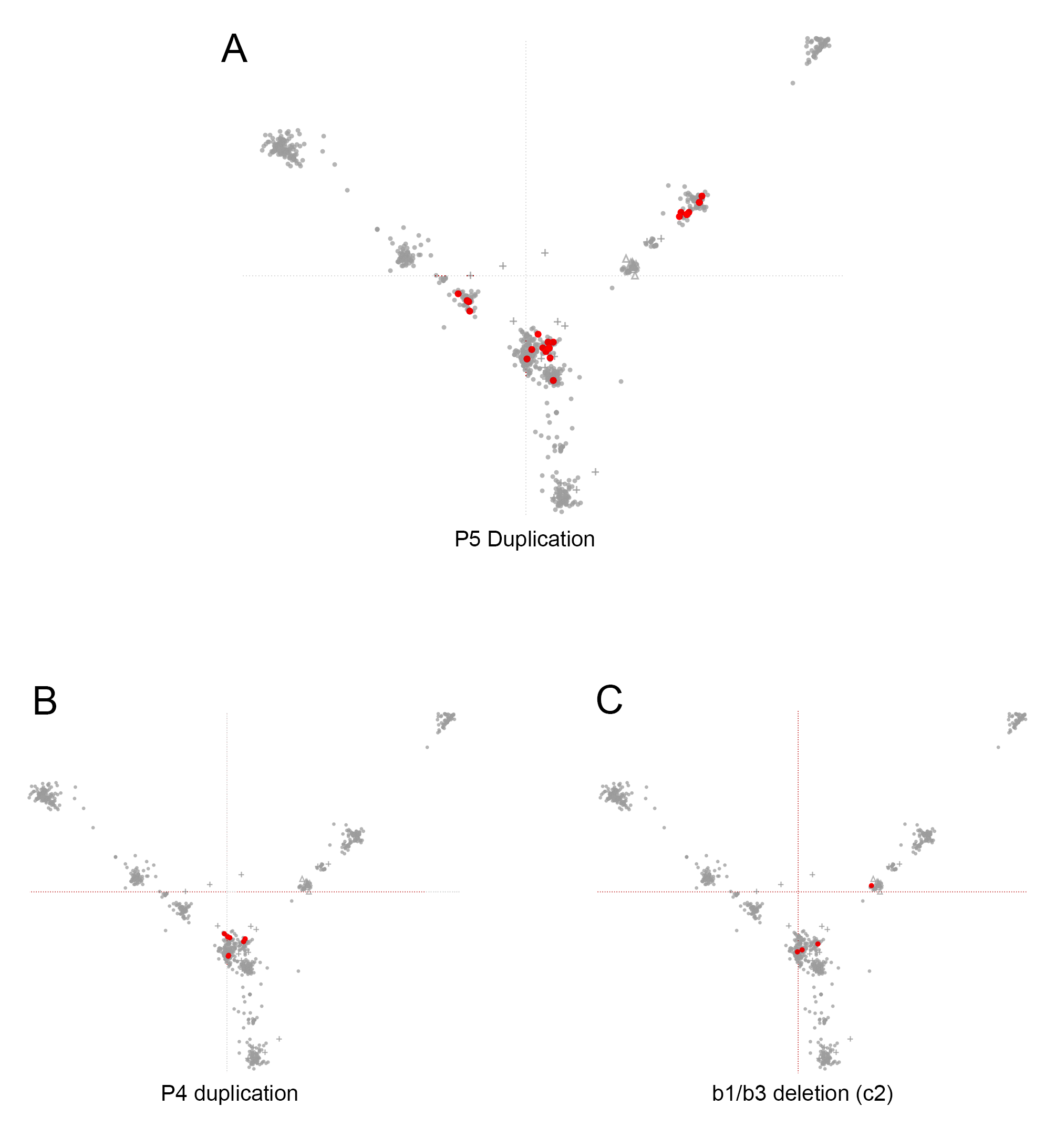

Supplement: S3 Fig — Each part of the Fig shows the graphical representation of the first two eigenvectors after PCA analysis. A: The Fig shows PCA values for individuals with P5 duplications significantly overrepresented in haplogroups E-M96, G-M201 and QM242. B: Individuals with P4 duplications significantly overrepresented in haplogroups KLT-M9(xM526) and Q-M242. C: Individuals with b1/b3 deletions (c2) significantly overrepresented in haplogroups C-M130 and O-M175. (TIF) [file pone.0137223.s003.tif]
