# Supplementary material for: Microarray Analysis of Copy Number Variants on the Human Y Chromosome Reveals Novel and Frequent Duplications Overrepresented in Specific Haplogroups
Source: PLoS One. 2015 Aug 31;10(8):e0137223. doi: 10.1371/journal.pone.0137223 (PMC4554990; doi:10.1371/journal.pone.0137223)
Supplement: S1 Table — The table shows all datasets that are included in this study. All male samples available as well as the samples that could be assigned to a HG and included into the total set of 1506 individuals are displayed in the two first columns. Number of deletions, duplications and both deletion and duplication patterns are displayed for included samples and the all samples together with the corresponding percentage frequencies. Last column displays the overall percentages of CNVs for each study, given for both included and all samples. (DOCX) [file pone.0137223.s004.docx]

Supplementary Table 1. All datasets included in the study and CNV frequencies within each dataset

| All samples | Samples haplogrouped and included | Dataset | Deletion | Duplication | Both Del Dupl | Del % | Dupl % | Both Del Dupl | Overall CNV frequency |
| --- | --- | --- | --- | --- | --- | --- | --- | --- | --- |
| 271 |  | Norwegian all samples | 15 | 15 | 1 | 5.5% | 5.5% | 0.4% | 11.4% |
|  | 176 | Norwegian haplogrouped | 13 | 11 | 1 | 7.4% | 6.3% | 0.6% | 14.2% |
| 11 |  | GSE21661 Tibetan all samples | 0 | 2 | 0 | 0.0% | 18.2% | 0.0% | 18.2% |
|  | 3 | GSE21661 Tibetan haplogrouped | 0 | 2 | 0 | 0.0% | 66.7% | 0.0% | 66.7% |
| 47 |  | GSE29851 Bolivian all sampels | 1 | 9 | 0 | 2.1% | 19.1% | 0.0% | 21.3% |
|  | 47 | GSE29851 Bolivian haplogrouped | 1 | 9 | 0 | 2.1% | 19.1% | 0.0% | 21.3% |
| 79 |  | GSE30481 Chinese all sampels | 10 | 10 | 0 | 12.7% | 12.7% | 0.0% | 25.3% |
|  | 65 | GSE30481 Chinese haplogrouped | 6 | 9 | 0 | 9.2% | 13.8% | 0.0% | 23.1% |
| 44 |  | GSE18333 Prostate all samples | 1 | 3 | 0 | 2.3% | 6.8% | 0.0% | 9.1% |
|  | 43 | GSE18333 Prostate haplogrouped | 1 | 3 | 0 | 2.3% | 7.0% | 0.0% | 9.3% |
| 415 |  | GSE23201 Schizophrenia all samples | 14 | 37 | 2 | 3.4% | 8.9% | 0.5% | 12.8% |
|  | 395 | GSE23201 Schizophrenia haplogrouped | 14 | 36 | 2 | 3.5% | 9.1% | 0.5% | 13.2% |
| 236 |  | GSE23636 Ashkenazi all samples | 3 | 23 | 0 | 1.3% | 9.7% | 0.0% | 11.0% |
|  | 215 | GSE23636 Ashkenazi haplogrouped | 2 | 22 | 0 | 0.9% | 10.2% | 0.0% | 11.2% |
| 77 |  | GSE15826 Motor neuron all samples | 1 | 7 | 0 | 1.3% | 9.1% | 0.0% | 10.4% |
|  | 71 | GSE15826 Motor neuron haplogrouped | 1 | 5 | 0 | 1.4% | 7.0% | 0.0% | 8.5% |
| 5 |  | GSE13429 Colorectal cancer all samples | 0 | 0 | 0 | 0.0% | 0.0% | 0.0% | 0.0% |
|  | 3 | GSE13429 Colorectal cancer haplogrouped | 0 | 0 | 0 | 0.0% | 0.0% | 0.0% | 0.0% |
| 510 |  | HapMap3 all samples | 20 | 48 | 6 | 3.9% | 9.4% | 1.2% | 14.5% |
|  | 475 | HapMap3 haplogrouped | 18 | 46 | 6 | 3.8% | 9.7% | 1.3% | 14.7% |
| 23 |  | CG-SER-426 developmental all samples | 12 | 1 | 1 | 52.2% | 4.3% | 4.3% | 60.9% |
|  | 13 | CG-SER-426 developmental haplgrouped | 12 | 0 | 1 | 92.3% | 0.0% | 7.7% | 100.0% |
| 1718 | 1506 |  |  |  |  |  |  |  |  |
