# Supplementary material for: Microarray Analysis of Copy Number Variants on the Human Y Chromosome Reveals Novel and Frequent Duplications Overrepresented in Specific Haplogroups
Source: PLoS One. 2015 Aug 31;10(8):e0137223. doi: 10.1371/journal.pone.0137223 (PMC4554990; doi:10.1371/journal.pone.0137223)
Supplement: S2 Table — The table includes the names, chromosome position and the average intensity values generated by surveillance of 61 females for SNP/CN probes with high intensity values in females compared to males. Log2R average values for three consecutive probes are also shown, since this is the minimum amount of probes that can be used in CNV detection by the Affymetrix Genotyping Console Software 4.1.3.840 (GTC). Three regions exhibited high average values (above -0.51) rendering them unsuitable for CNV detection in males. (DOCX) [file pone.0137223.s005.docx]

### Supplementary Table 2 – SNP/CN probes in MSY exhibiting high intensity values based on hybridization of 61 female samples

| **Probe Id** | **Chromosome position** | **Log2R average (61 females)** | **Log2R average for 3 probes** |
| --- | --- | --- | --- |
| CN_950326 | 4684219 | -0.75 |  |
| CN_950327 | 4688110 | -0.14 | -0.36 |
| SNP_A-8289973 | 4696023 | -0.18 |  |
| CN_952581 | 5364058 | -0.69 |  |
| SNP_A-8380633 | 5364159 | -0.29 | -0.47 |
| CN_952582 | 5365043 | -0.43 |  |
| SNP_A-8504756 | 5916713 | -0.70 |  |
| SNP_A-8568779 | 5926628 | -0.61 | -0.40 |
| SNP_A-8697464 | 5943453 | 0.11 |  |
