# Supplementary material for: Microarray Analysis of Copy Number Variants on the Human Y Chromosome Reveals Novel and Frequent Duplications Overrepresented in Specific Haplogroups
Source: PLoS One. 2015 Aug 31;10(8):e0137223. doi: 10.1371/journal.pone.0137223 (PMC4554990; doi:10.1371/journal.pone.0137223)
Supplement: S3 Table — The table shows the distribution of CNV patterns among haplogroups for 15 variants that did not show overrepresentation in any group. Ambiguous individuals, for which haplotype determination was not possible, are shown in the table for completeness, but they were not included in the statistical analysis. Two individuals exhibited CNV patterns that were not possible to classify into any variant category, and they are therefore listed as “unspecified”. (DOCX) [file pone.0137223.s006.docx]

**Supplementary Table 3. Distribution of CNV patterns and their frequency within haplogroups**

| **Haplogroup** | **Nr of individuals** | **P-arm dupl** | **Q-arm del** | **P6 dupl** | **Prior P5 post P4 dupl** | **P5 del** | **IR2 dupl** | **IR2 del** | **P3 del** | **b2/b4 del dupl c6** | **b2/b4 dupl c21** | **b2/b4 dupl c56** | **gr/gr dupl c9** | **Y1Y2 dupl** | **Distal gr dupl** | **Distal gr del** | **Unspeci- fied** |
| --- | --- | --- | --- | --- | --- | --- | --- | --- | --- | --- | --- | --- | --- | --- | --- | --- | --- |
| **C-M130** | 24 |  |  |  |  |  |  |  |  |  |  |  | 1 |  |  |  |  |
| **D-M174** | 6 |  |  |  |  |  |  |  |  |  |  |  |  |  |  |  |  |
| **E-M96** | 339 | 1 |  |  | 7 | 1 | 9 |  | 5 | 3 | 2 |  | 3 |  |  | 3 |  |
| **F-M89(xM1329)** | 10 |  |  |  |  |  |  |  |  |  |  |  |  |  |  |  |  |
| **G-M201** | 80 |  |  |  |  |  |  |  |  |  |  |  | 2 |  |  |  | 1 |
| **I-M170** | 40 |  |  |  |  |  |  |  |  |  | 1 |  | 5 |  |  |  |  |
| **J-M304** | 242 |  |  |  |  |  |  |  |  | 1 | 1 |  | 8 | 1 | 1 |  |  |
| **KLT-M9(xM526)** | 29 | 1 |  |  |  |  |  |  |  |  |  |  |  |  |  |  |  |
| **NO-M214(xM175)** | 40 |  |  | 1 |  |  |  |  |  |  |  |  |  |  |  |  | 1 |
| **O-M175** | 162 |  |  |  |  |  |  | 1 |  | 3 |  | 1 | 5 |  |  |  |  |
| **Q-M242** | 56 |  |  |  |  |  | 1 |  |  |  | 2 |  | 3 |  |  |  |  |
| **R-M207** | 478 | 3 |  | 5 |  | 1 | 8 |  |  |  | 1 | 1 | 11 |  |  | 2 |  |
| Ambiguous |  |  | 1 |  |  |  |  |  |  |  |  |  | 1 |  |  |  |  |
| Sum of CNV type |  | 5 | 0 | 6 | 7 | 2 | 18 | 1 | 5 | 7 | 7 | 2 | 38 | 1 | 1 | 5 | 2 |
| % of CNV type |  | 0.33% | 0.00% | 0.40% | 0.46% | 0.13% | 1.20% | 0.07% | 0.33% | 0.46% | 0.46% | 0.13% | 2.52% | 0.07% | 0.07% | 0.33% | 0.13% |
